# Lipid remodeling enables adaptation to chronic hyperosmotic stress

**DOI:** 10.1083/jcb.202603154

**Published:** 2026-10-08

**Authors:** Thomas D. Williams, Christian Klose, Robert Ernst, Pedro Carvalho

**Affiliations:** 1 Sir William Dunn School of Pathology, University of Oxford, Oxford, UK; 2 Lipotype GmbH, Dresden, Germany; 3 Medical Biochemistry and Molecular Biology, Saarland University, Homburg, Germany; 4 Preclinical Center for Molecular Signaling, Saarland University, Homburg, Germany

## Abstract

Lipid droplets (LDs) accumulate in response to diverse cellular stresses. However, their regulation and physiological roles remain poorly understood in most contexts. Here, we show that in budding yeast, chronic hyperosmotic stress induces sustained LD accumulation. Unlike the transient LD response observed during acute osmotic shock, chronic stress triggers prolonged, Dga1-dependent triacylglycerol synthesis. In the absence of triacylglycerol synthesis, cellular fitness is impaired. Lipidomic profiling reveals extensive membrane remodeling during chronic hyperosmotic stress, most notably an increase in phosphatidylcholine (PC) resulting in changes to membrane properties. In LD-deficient cells, the hyperosmotic stress–induced PC increase is blunted, while enhancing PC synthesis improves the fitness of triacylglycerol-deficient cells. Thus, LD accumulation and phospholipid remodeling underlie an adaptive response to chronic hyperosmotic stress.

## Teaser

How cells adapt their membranes to prolonged stress is poorly understood. Williams et al. show that chronic hyperosmotic stress triggers lipid droplet accumulation and a shift toward phosphatidylcholine in budding yeast. Triacylglycerol synthesis supports adaptive changes in membrane properties, while elevated phosphatidylcholine promotes cell fitness.

## Introduction

Lipid droplets (LDs) are unique lipid storage organelles present in most eukaryotic cells (Mathiowetz and Olzmann, 2024; Farese and Walther, 2025; Klemm and Carvalho, 2024). They are composed of a phospholipid monolayer surrounding a hydrophobic core of neutral lipids, primarily triacylglycerols (TAGs) and sterol esters (STEs). TAGs are synthesized through the acylation of diacylglycerol (DAG) with a fatty acid, either derived from an activated acyl-CoA or transferred from a donor phospholipid (Athenstaedt, 2010). STEs are generated by esterifying sterols with acylated fatty acids (Athenstaedt, 2010). LD biogenesis occurs by an evolutionarily conserved mechanism in which seipin oligomers concentrate neutral lipids within the ER bilayer, thereby facilitating their condensation into lens structures that subsequently bud toward the cytoplasm to originate a mature LD (Klug et al., 2024; Thiam and Ikonen, 2021).

Proper LD assembly and regulation are essential for cellular and organismal health by maintaining membrane homeostasis and energy balance (Zadoorian et al., 2023). Defects in LD formation result in lipodystrophies, best exemplified by the seipinopathies (Cartwright and Goodman, 2012), while excessive LD formation is the defining feature of fatty liver disease (Heeren and Scheja, 2021) and is further associated with poor cancer prognosis (Quan et al., 2025). Because many environmental stresses perturb membrane composition and biophysical properties, LD accumulation is a common adaptive response (Pressly et al., 2022; Jin et al., 2023; Safi et al., 2024). Lipids stored in LDs contribute to ATP synthesis and cell adaptation to nutrient limitation (Seo et al., 2017; Rambold et al., 2015; Hariri et al., 2018; Rogers et al., 2021). When membrane expansion is required, such as upon growth resumption from quiescence, LDs can be rapidly turned over to supply lipids for membrane biogenesis (Markgraf et al., 2014). Conversely, recent evidence shows that LDs can buffer excess lipids and membranes (Phan et al., 2025; Wang et al., 2018) and sequester membrane lipids that are susceptible to oxidation (Lange et al., 2025), or otherwise compromise organelle integrity (Nguyen and Olzmann, 2017). Fluctuations in temperature alter membrane properties (Ernst et al., 2016), and the inability to make LDs renders cells sensitive to changes in temperature (Wang et al., 2018). Together, these findings suggest that LDs enable cells to remodel membrane composition and maintain membrane homeostasis under stress conditions.

Here, building on a high-throughput approach to quantify LD abundance in budding yeast, we identified a previously unrecognized LD response to chronic hyperosmotic stress that is required for cellular fitness. Recent reports have demonstrated rapid and transient LD accumulation upon acute (<30 min) hyperosmotic stress (Phan et al., 2025), but longer term adaptations of membranes and organelles to allow cell proliferation are less well studied. We show that LD accumulation is important for proliferation under sustained, chronic (>3 h) hyperosmotic stress. This response occurs in parallel to, but is not directly controlled by, the canonical hyperosmotic adaptation machinery and is necessary for modulating membrane properties. LD accumulation is accompanied by increased phosphatidylcholine (PC) levels, an increase that is blunted in the absence of LDs. Promoting PC synthesis alleviates the requirement for LD formation during chronic hyperosmotic stress. Together, our findings identify LD accumulation as a key adaptive response to chronic hyperosmotic stress, contributing to membrane remodeling and supporting cellular fitness.

## Results

**Figure S1.**
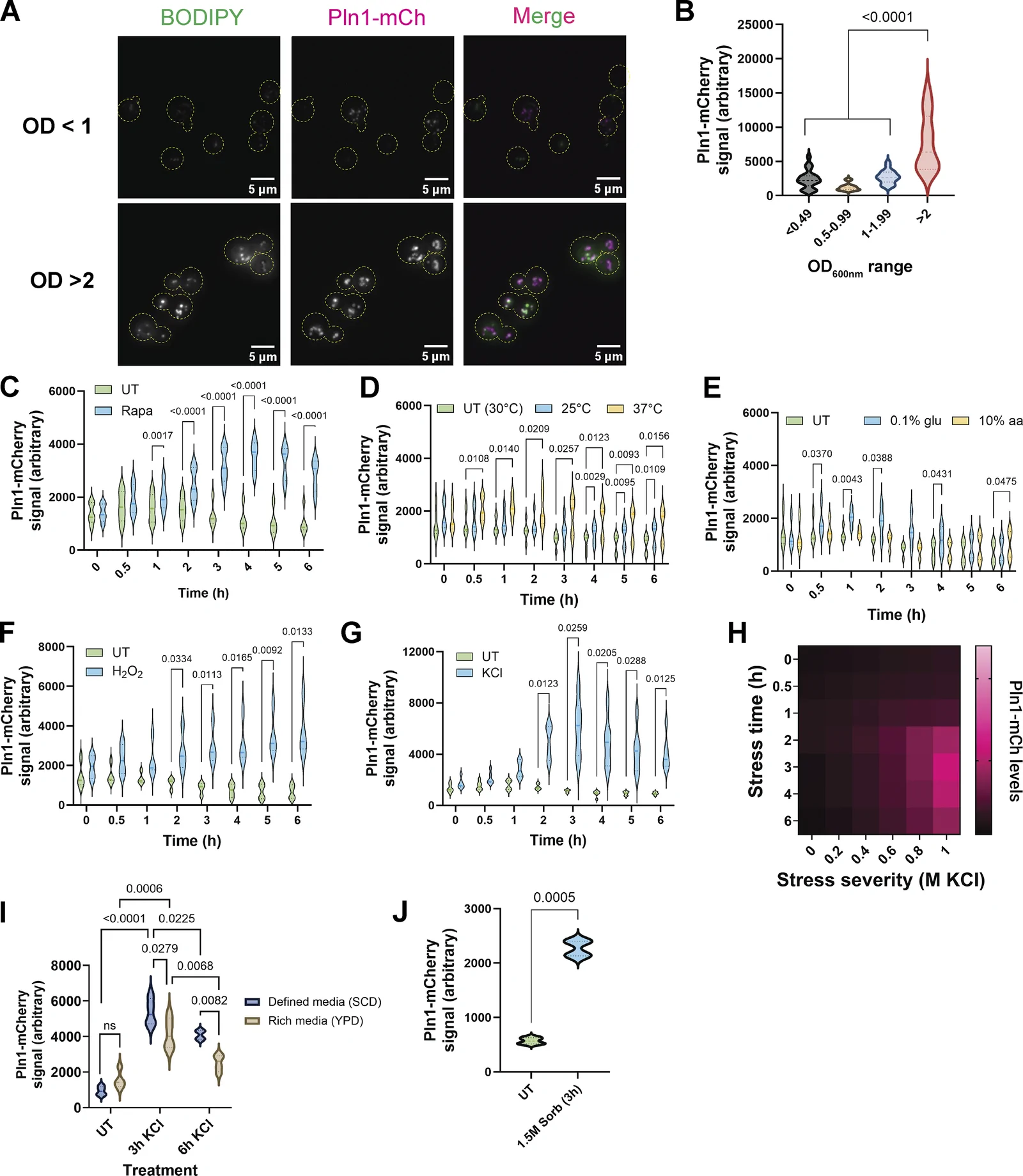
**Screening of LD responses to stress. (A)** Pln1-mCherry colocalization with BODIPY-stained LDs in exponential (OD < 1)- and stationary-phase (OD > 2) cells. Z-projection images are representative of multiple replicates. **(B)** Median Pln1-mCherry fluorescence measured by flow cytometry from populations of single WT cells grown to varying densities, measured and grouped by the indicated ranges of absorbance at OD_600 nm_. Violin plots show the spread of data, with the dashed lines indicating the median and the dotted lines the upper and lower quartiles. *N* = 22 (<0.49), 7 (0.5–0.99), 19 (1–1.99), 12 (>2). **(C)** Median Pln1-mCherry fluorescence measured by flow cytometry from populations of single WT cells treated or not with 200 µM rapamycin for the indicated time points. Violin plots show the spread of data, with the dashed lines indicating the median and the dotted lines the upper and lower quartiles. *N* = 5. **(D)** Median Pln1-mCherry fluorescence measured by flow cytometry from populations of single WT cells subjected to the indicated temperature shifts from 30°C for the indicated time points. Violin plots show the spread of data, with the dashed lines indicating the median and the dotted lines the upper and lower quartiles. *N* = 5. **(E)** Median Pln1-mCherry fluorescence measured by flow cytometry from populations of single WT cells subjected to the indicated nutrient restriction for the indicated time points. Violin plots show the spread of data, with the dashed lines indicating the median and the dotted lines the upper and lower quartiles. *N* = 4. **(F)** Median Pln1-mCherry fluorescence measured by flow cytometry from populations of single WT cells subjected to 4 mM H_2_O_2_ exposure for the indicated time points. Violin plots show the spread of data, with the dashed lines indicating the median and the dotted lines the upper and lower quartiles. *N* = 4. **(G)** Median Pln1-mCherry fluorescence measured by flow cytometry from populations of single WT cells subjected to 1M KCl hyperosmotic stress for the indicated time points. Violin plots show the spread of data, with the dashed lines indicating the median and the dotted lines the upper and lower quartiles. *N* = 6. **(H)** Median Pln1-mCherry fluorescence measured by flow cytometry from populations of single WT cells subjected to varying times and severities of KCl treatment in SCD media. UT = untreated. The color scale indicates the mean values from three biological replicates. **(I)** Median Pln1-mCherry fluorescence measured by flow cytometry from populations of single WT cells in either SCD or YPD subjected to 1M KCl treatment for the indicated times. UT = untreated. Violin plots show the spread of data, with the dashed lines indicating the median and the dotted lines the upper and lower quartiles. *N* = 4. **(J)** Median Pln1-mCherry fluorescence measured by flow cytometry from populations of single WT cells subjected to 1.5M sorbitol treatment in SCD media for 3 h. UT = untreated. Violin plots show the spread of data, with the dashed lines indicating the median and the dotted lines the upper and lower quartiles. *N* = 4.

Pln1 localizes to the surface of LDs and is rapidly degraded when not bound to LDs (Gao et al., 2017). Fluorescently tagged Pln1 is therefore commonly used as an LD marker (Fig. S1 A) (Gao et al., 2017; Giménez-Andrés et al., 2021; Wang et al., 2024), and its levels are a good surrogate for LD abundance (Rao et al., 2025, *Preprint*; Gao et al., 2017). To investigate LD accumulation dynamics in a high-throughput manner, we measure the levels of endogenously tagged Pln1-mCherry using flow cytometry. During exponential growth, Pln1-mCherry levels sharply increase as cells approach the stationary phase, mirroring LD abundance as detected by fluorescence microscopy of BODIPY-stained cells (Fig. S1, A and B).

To further validate the flow cytometry–based results, we used pharmacological inhibition of TORC1 with rapamycin, a treatment known to induce LD accumulation (Madeira et al., 2015; Teixeira et al., 2021; Liu et al., 2019; Hosios et al., 2022; Dubots et al., 2014). As expected, rapamycin treatment of wild-type (WT) cells resulted in a rapid and sustained increase of Pln1-mCherry levels (Fig. S1 C). Therefore, flow cytometry analysis of Pln1-mCherry levels provides a sensitive and accurate readout of LD accumulation.

### Chronic hyperosmotic stress induces LD accumulation

**Figure 1.**
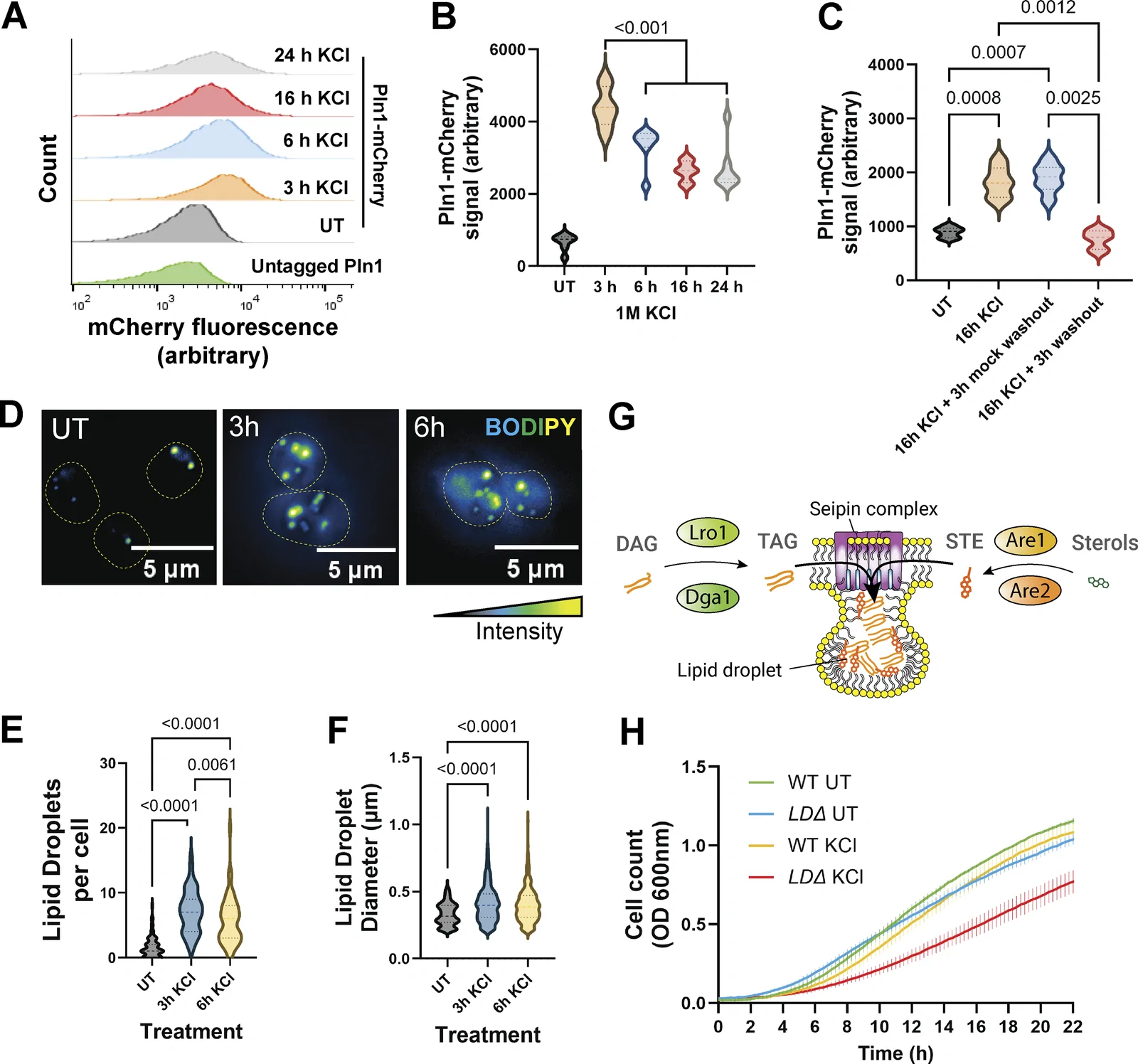
**LD accumulation is a chronic response to hyperosmotic stress. (A)** Representative histograms obtained from 10,000 Pln1-mCherry–tagged cells grown in SCD + 1M KCl for the indicated time points. The untagged Pln1 shows the background fluorescence, while UT is untreated. **(B)** Median Pln1-mCherry fluorescence measured by flow cytometry from populations of single cells subjected to 1M KCl treatment in SCD media for the indicated time. UT = untreated. Violin plots show the spread of data, with the dashed lines indicating the median and the dotted lines the upper and lower quartiles. *N* = 7. **(C)** Median Pln1-mCherry fluorescence measured by flow cytometry from populations of single cells subjected to 16 h of 1M KCl treatment in SCD media which was then washed out and replaced with SCD + 1M KCl (mock washout) or SCD without 1M KCl for 3 h before measurement. UT = untreated. Violin plots show the spread of data, with the dashed lines indicating the median and the dotted lines the upper and lower quartiles. *N* = 6. **(D)** LDs stained with BODIPY and imaged by live-cell microscopy after varying lengths of 1M KCl treatment in SCD. Z-projection images are representative of multiple replicates. UT = untreated. **(E)** Analysis of LDs per cell from samples used in C. UT = untreated. Violin plots show the spread of data, with the dashed lines indicating the median and the dotted lines the upper and lower quartiles. *N* = 85 (UT), 89 (3 h KCl), 64 (6 h KCl) cells from three biological replicates. **(F)** Analysis of LD diameters from samples used in C. UT = untreated. Violin plots show the spread of data, with the dashed lines indicating the median and the dotted lines the upper and lower quartiles. *N* = 165 (UT), 626 (3 h KCl), 427 (6 h KCl) LDs from three biological replicates. **(G)** Schematic of LD formation. Dga1 or Lro1 converts DAG into TAG, while Are1 or Are2 converts sterols into STEs. TAGs and STEs are concentrated and packaged into LDs by the seipin complex. **(H)** Growth assay of WT and a*re1Δare2Δdga1Δlro1Δ* (*LDΔ*) cells in SCD and SCD + 1M KCl. The main line shows the mean, with the error bars showing SEM from four biological replicates.

We next monitored the levels of Pln1-mCherry over time in exponentially growing cells subjected to a variety of stresses: oxidative, temperature, nutrient, and hyperosmotic. Upon moderate temperature shifts (from 30 to 25°C or 37°C) or acute restriction of glucose or amino acids (2–0.1% glucose or a 10-fold reduction in amino acid levels), cells showed minimal changes in Pln1-mCherry levels over a 6-h period (Fig. S1, D and E). Oxidative stress induced by H_2_O_2_ resulted in a mild and gradual Pln1-mCherry accumulation (Fig. S1 F). In contrast, hyperosmotic stress induced by KCl resulted in a rapid increase in Pln1-mCherry accumulation that peaked at 3 h after stress induction and remained high for at least 24 h (Fig. 1, A and B; and Fig. S1 G). Moreover, the increase in Pln1-mCherry levels triggered by KCl-induced hyperosmotic stress was dose- and time-dependent (Fig. S1 H), and occurred in both rich and defined media (Fig. S1 I). Moreover, a similar increase in Pln1-mCherry levels was observed when cells were grown in sorbitol, a nonionic osmolyte (Fig. S1 J). Importantly, a reduction of Pln1-mCherry to baseline levels was observed within 3 h upon removal of the hyperosmotic stress showing this response was reversible (Fig. 1 C). Finally, we confirmed that the increase in Pln1-mCherry levels upon hyperosmotic stress was due to LD accumulation by imaging of BODIPY-labeled cells (Fig. 1 D). This analysis showed that KCl treatment increased both the number and size of LDs in cells (Fig. 1, E and F). Together, these results demonstrate that chronic hyperosmotic stress triggers long-lasting but reversible LD accumulation.

### LD accumulation is important for cell fitness under hyperosmotic stress

Hyperosmotic stress causes rapid water egress and a drop in plasma membrane tension (Roffay et al., 2021; Phan et al., 2025). The drop in plasma membrane tension leads to acute responses to hyperosmotic stress. These include temporary LD accumulation, dependent upon efficient endocytosis and the lipid transfer proteins Osh2 and Osh3 (Phan et al., 2025). Plasma membrane tension is, however, typically restored within 30–60 min of stress onset (Klipp et al., 2005; Shen et al., 2023; Petelenz-Kurdziel et al., 2013), long before the phenotypes we observe. We therefore hypothesized that LD accumulation may be important for long-term fitness under persistent hyperosmotic stress, beyond its role in restoring plasma membrane tension.

**Figure S2.**
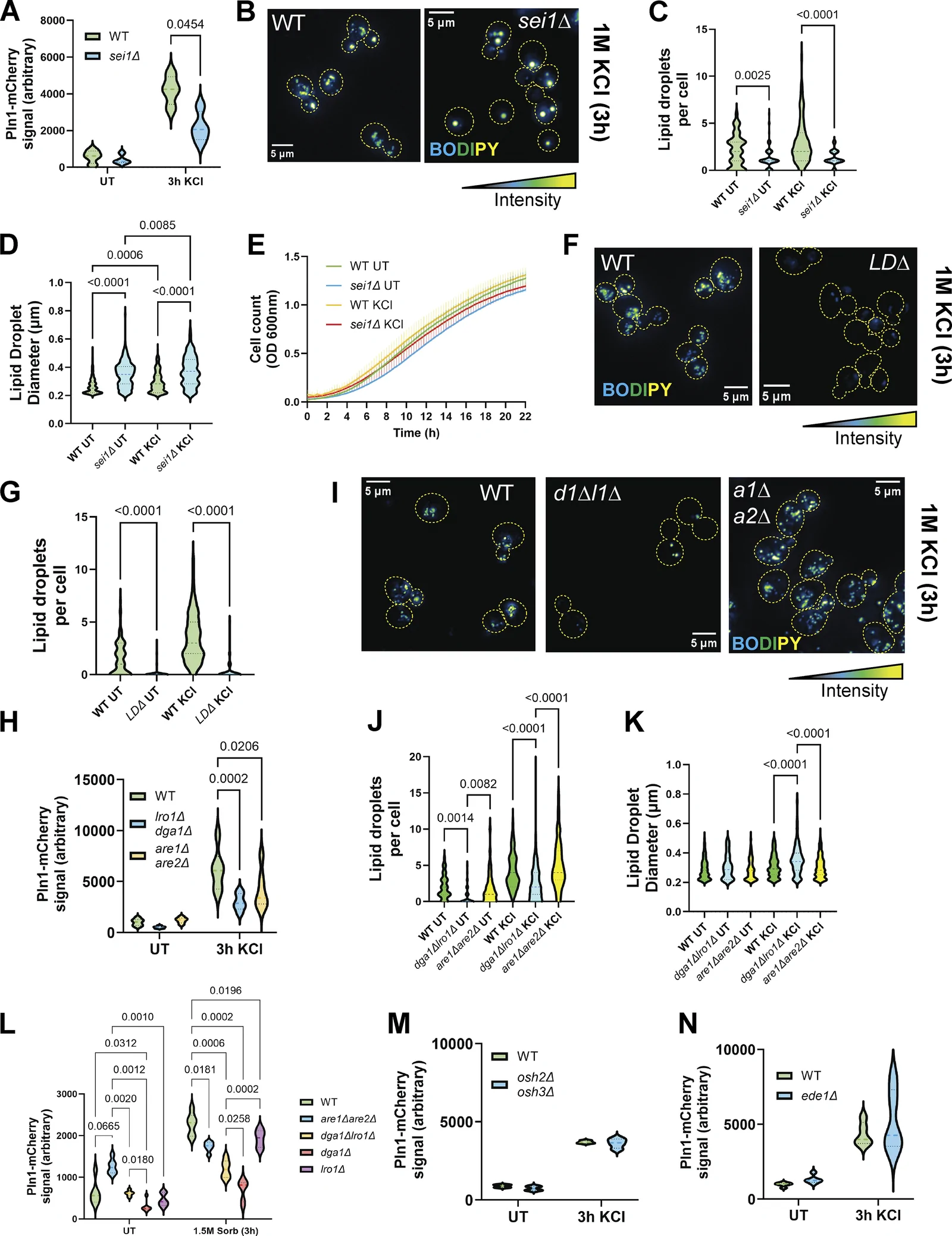
**LD responses to hyperosmotic stress. (A)** Median Pln1-mCherry fluorescence measured by flow cytometry from populations of single WT and *sei1Δ* cells subjected to 1M KCl treatment in SCD media for 3 h. UT = untreated. Violin plots show the spread of data, with the dashed lines indicating the median and the dotted lines the upper and lower quartiles. *N* = 4. **(B)** LDs stained with BODIPY and imaged by live-cell microscopy after 3 h of 1M KCl treatment in SCD for Pln1-mCherry WT and *sei1Δ* cells. Z-projection images are representative of multiple replicates. **(C)** Analysis of LDs per cell from samples used in B. UT = untreated. Violin plots show the spread of data, with the dashed lines indicating the median and the dotted lines the upper and lower quartiles. *N* = 95 (WT UT), 107 (*sei1Δ* UT), 104 (WT KCl), 92 (*sei1Δ* KCl) cells from three biological replicates. **(D)** Analysis of LD diameters from samples used in B. UT = untreated. Violin plots show the spread of data, with the dashed lines indicating the median and the dotted lines the upper and lower quartiles. *N* = 251 (WT UT), 276 (*sei1Δ* UT), 253 (WT KCl), 230 (*sei1Δ* KCl) LDs from three biological replicates. **(E)** Growth assay of WT and *sei1Δ* cells in SCD and SCD + 1M KCl. The graph shows the mean and SEM from four biological replicates. **(F)** LDs stained with BODIPY and imaged by live-cell microscopy after 3 h of 1M KCl treatment in SCD for WT and a*re1Δare2Δdga1Δlro1Δ* (*LDΔ*) cells. Z-projection images are representative of multiple replicates. **(G)** Analysis of LDs per cell from samples in F. UT = untreated. Violin plots show the spread of data, with the dashed lines indicating the median and the dotted lines the upper and lower quartiles. *N* = 103 (WT UT), 96 (*LDΔ* UT), 97 (WT KCl), 108 (*LDΔ* KCl) cells from three biological replicates. **(H)** Median Pln1-mCherry fluorescence measured by flow cytometry from populations of single WT, *dga1Δlro1Δ*, and *are1Δare2Δ* cells subjected to 1M KCl treatment in SCD media for 3 h. UT = untreated. Violin plots show the spread of data, with the dashed lines indicating the median and the dotted lines the upper and lower quartiles. *N* = 6. **(I)** LDs stained with BODIPY and imaged by live-cell microscopy after 3 h of 1M KCl treatment in SCD for Pln1-mCherry WT, *dga1Δlro1Δ*, and *are1Δare2Δ* cells. Z-projection images are representative of multiple replicates. **(J)** Analysis of LDs per cell from samples in I. UT = untreated. Violin plots show the spread of data, with the dashed lines indicating the median and the dotted lines the upper and lower quartiles. *N* = 124 (WT UT), 121 (*dga1Δlro1Δ* UT), 125 (*are1Δare2Δ* UT), 120 (WT KCl), 113 (*dga1Δlro1Δ* KCl), 78 (*are1Δare2Δ* KCl) cells from 4 biological replicates. **(K)** Analysis of LD diameters from samples in I. UT = untreated. Violin plots show the spread of data, with the dashed lines indicating the median and the dotted lines the upper and lower quartiles. *N* = 414 (WT UT), 173 (*dga1Δlro1Δ* UT), 250 (*are1Δare2Δ* UT), 504 (WT KCl), 218 (*dga1Δlro1Δ* KCl), 262 (*are1Δare2Δ* KCl) LDs from four biological replicates. **(L)** Median Pln1-mCherry fluorescence measured by flow cytometry from populations of single WT, *are1Δare2Δ*, *dga1Δlro1Δ*, *dga1Δ*, and *lro1Δ* cells subjected to 1.5M sorbitol treatment in SCD media for 3 h. UT = untreated. Violin plots show the spread of data, with the dashed lines indicating the median and the dotted lines the upper and lower quartiles. *N* = 6. **(M)** Median Pln1-mCherry fluorescence measured by flow cytometry from populations of single WT and *osh2Δosh3Δ* cells subjected to 1M KCl treatment in SCD media for 3 h. UT = untreated. Violin plots show the spread of data, with the dashed lines indicating the median and the dotted lines the upper and lower quartiles. *N* = 4. **(N)** Median Pln1-mCherry fluorescence measured by flow cytometry from populations of single WT and *ede1Δ* cells subjected to 1M KCl treatment in SCD media for 3 h. UT = untreated. Violin plots show the spread of data, with the dashed lines indicating the median and the dotted lines the upper and lower quartiles. *N* = 5.

To test this hypothesis, we examined mutants defective in LD biogenesis. LD biogenesis depends on neutral lipid synthesis and is spatially controlled by the seipin complex (Fig. 1 G). Seipin mutants produce aberrant LDs that are defective in recruiting surface proteins including Pln1-mCherry (Grippa et al., 2015). Consistent with this, seipin mutants showed reduced Pln1-mCherry accumulation under hyperosmotic stress (Fig. S2 A), and most cells had only one or two large LDs (Fig. S2, B and C). Since the existing LDs expanded under stress, neutral lipids were still produced (Fig. S2 D). Of note, the seipin-dependent changes in LD size did not compromise cell fitness under hyperosmotic conditions (Fig. S2 E). We next tested the importance of stress-induced neutral lipid synthesis using a strain that lacks all four relevant enzymes (Dga1 and Lro1, which produce TAGs, and Are1 and Are2, which synthesize STEs, referred to here as *LD*Δ) (Oelkers et al., 2002; Sandager et al., 2002) and consequently is devoid of LDs (Fig. S2, F and G). Unlike seipin mutants, *LDΔ* cells exhibited a pronounced growth defect under hyperosmotic stress (Fig. 1 H). Together, these findings indicate that a process related to LD accumulation, rather than normal LD morphology per se, is required for adaptation to persistent hyperosmotic stress.

### Hyperosmotic LD accumulation is driven by Dga1-mediated TAG synthesis

**Figure 2.**
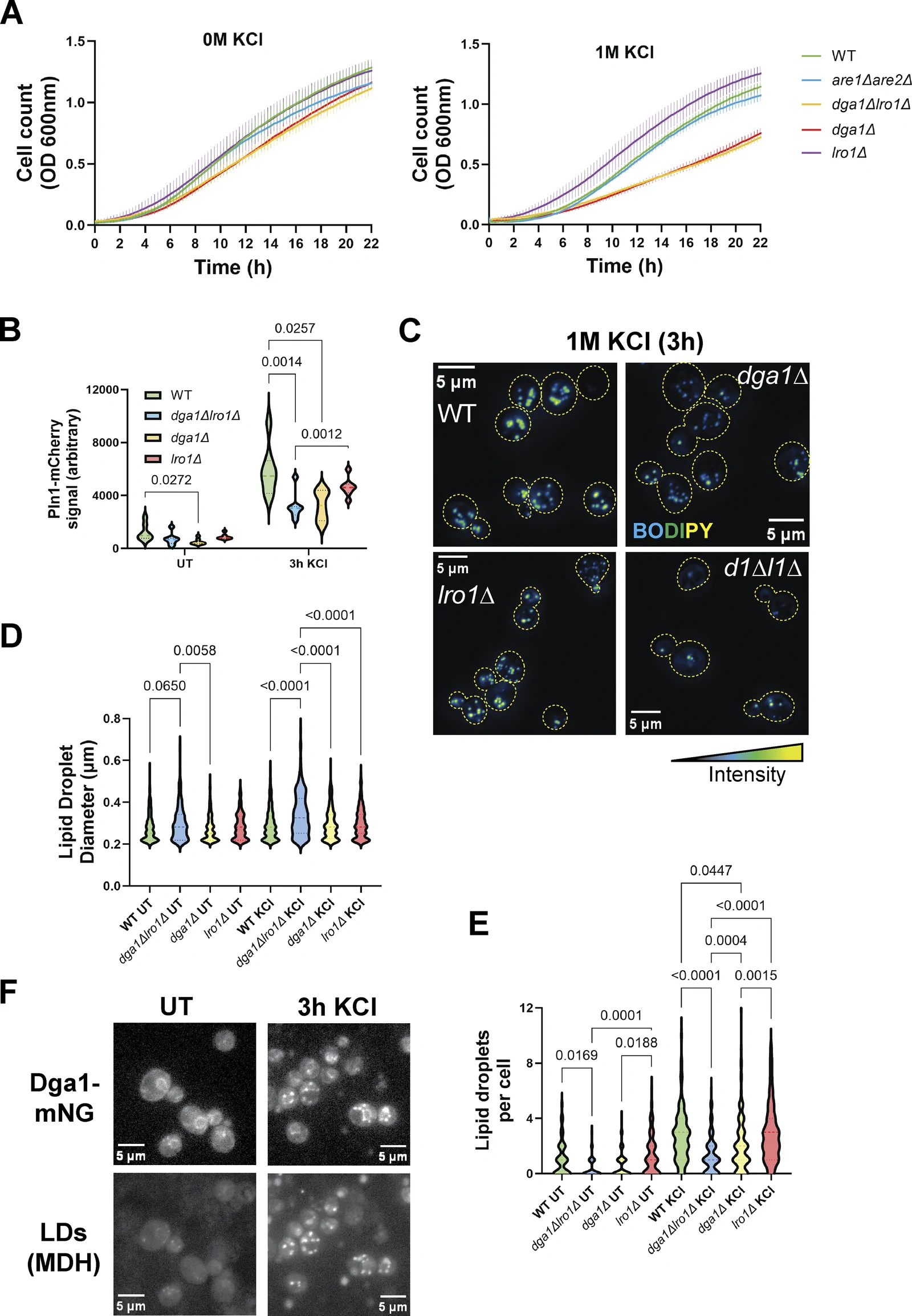
**Dga1-produced TAGs drive chronic hyperosmotic LD accumulation. (A)** Growth assay of WT, a*re1Δare2Δ* (*a1Δa2Δ*), *dga1Δlro1Δ* (*d1Δl1Δ*), *dga1Δ*, and *lro1Δ* cells in SCD and SCD + 1M KCl. Error bars show SEM from four biological replicates. **(B)** Median Pln1-mCherry fluorescence measured by flow cytometry from populations of single WT, *dga1Δlro1Δ, dga1Δ*, and *lro1Δ* cells subjected to 1M KCl treatment in SCD media for 3 h. UT = untreated. Violin plots show the spread of data, with the dashed lines indicating the median and the dotted lines the upper and lower quartiles. *N* = 7. **(C)** LDs stained with BODIPY and imaged by live-cell microscopy after 3 h of 1M KCl treatment in SCD for Pln1-mCherry WT, *dga1Δlro1Δ, dga1Δ*, and *lro1Δ* cells. Z-projection images are representative of multiple replicates. **(D)** Analysis of LDs per cell from samples used in C. UT = untreated. Violin plots show the spread of data, with the dashed lines indicating the median and the dotted lines the upper and lower quartiles. *N* = 124 (WT UT), 125 (*dga1Δlro1Δ* UT), 130 (*dga1Δ* UT), 108 (*lro1Δ* UT), 123 (WT KCl), 116 (*dga1Δlro1Δ* KCl), 135 (*dga1Δ* KCl), 115 (*lro1Δ* KCl) cells from four biological replicates. **(E)** Analysis of LD diameters from samples used in C. UT = untreated. Violin plots show the spread of data, with the dashed lines indicating the median and the dotted lines the upper and lower quartiles. *N* = 326 (WT UT), 141 (*dga1Δlro1Δ* UT), 379 (*dga1Δ* UT), 187 (*lro1Δ* UT), 514 (WT KCl), 275 (*dga1Δlro1Δ* KCl), 388 (*dga1Δ* KCl), 373 (*lro1Δ* KCl) LDs from four biological replicates. **(F)** Visualization of Dga1-mNG and LDs (stained using MDH) in live cells with and without 3 h of treatment with 1M KCl in SCD. UT = untreated. Images are representative of multiple replicates.

We next assessed the individual contribution of TAG and STE synthesis to cell fitness under hyperosmotic stress. Simultaneous deletion of *ARE1* and *ARE2*, which prevent STE synthesis, did not impact cell growth either in the absence or in the presence of hyperosmotic stress (Fig. 2 A). In contrast, preventing TAG synthesis with *dga1Δlro1Δ* cells resulted in a growth defect specifically under hyperosmotic stress (Fig. 2 A). Deletion of *DGA1*, but not *LRO1*, alone also resulted in a growth defect relative to the WT cells (Fig. 2 A). Therefore, TAG synthesis is required for adaptation to chronic hyperosmotic stress with Dga1 playing a major role.

Since the growth assays reveal the importance of TAG synthesis for long-term adaptation, we monitored Pln1-mCherry to investigate LD accumulation upon hyperosmotic stress. Consistent with the growth assays, *are1Δare2Δ* cells displayed no changes in LD levels compared with the WT cells using Pln1-mCherry or BODIPY as markers (Fig. S2, H–K). However, the LD response was strongly blunted in *dga1Δlro1Δ* cells (Fig. 2, B–E and Fig. S2, H−K). In contrast to acute hyperosmotic stress (Phan et al., 2025), Lro1 was dispensable for LD accumulation induced by chronic hyperosmotic stress (Fig. 2, B–E). On the other hand, *dga1Δ* cells showed much less Pln1-mCherry, comparable to that observed in *dga1Δlro1Δ* cells (Fig. 2 B). The predominant role of Dga1 was also seen when stress was induced with the nonionic osmolyte sorbitol (Fig. S2 L). The defect in LD accumulation in *dga1Δ* cells was further confirmed by imaging LDs after BODIPY labeling (Fig. 2, C–E). Therefore, Dga1 plays a major role in LD accumulation during chronic hyperosmotic stress, suggesting that LDs formed during chronic stress are regulated differently from those produced during the Lro1-dominated acute phase (Phan et al., 2025).

Supporting this being a distinct response, deletion of the endocytic protein Ede1 or the lipid transfer proteins Osh2 and Osh3, required for the acute phase LD formation (Phan et al., 2025), had no impact on Pln1-mCherry accumulation following 3 h of hyperosmotic stress (Fig. S2, M and N). This difference likely reflects distinct upstream cues and physiological functions during acute and chronic responses to hyperosmotic stress.

**Figure S3.**
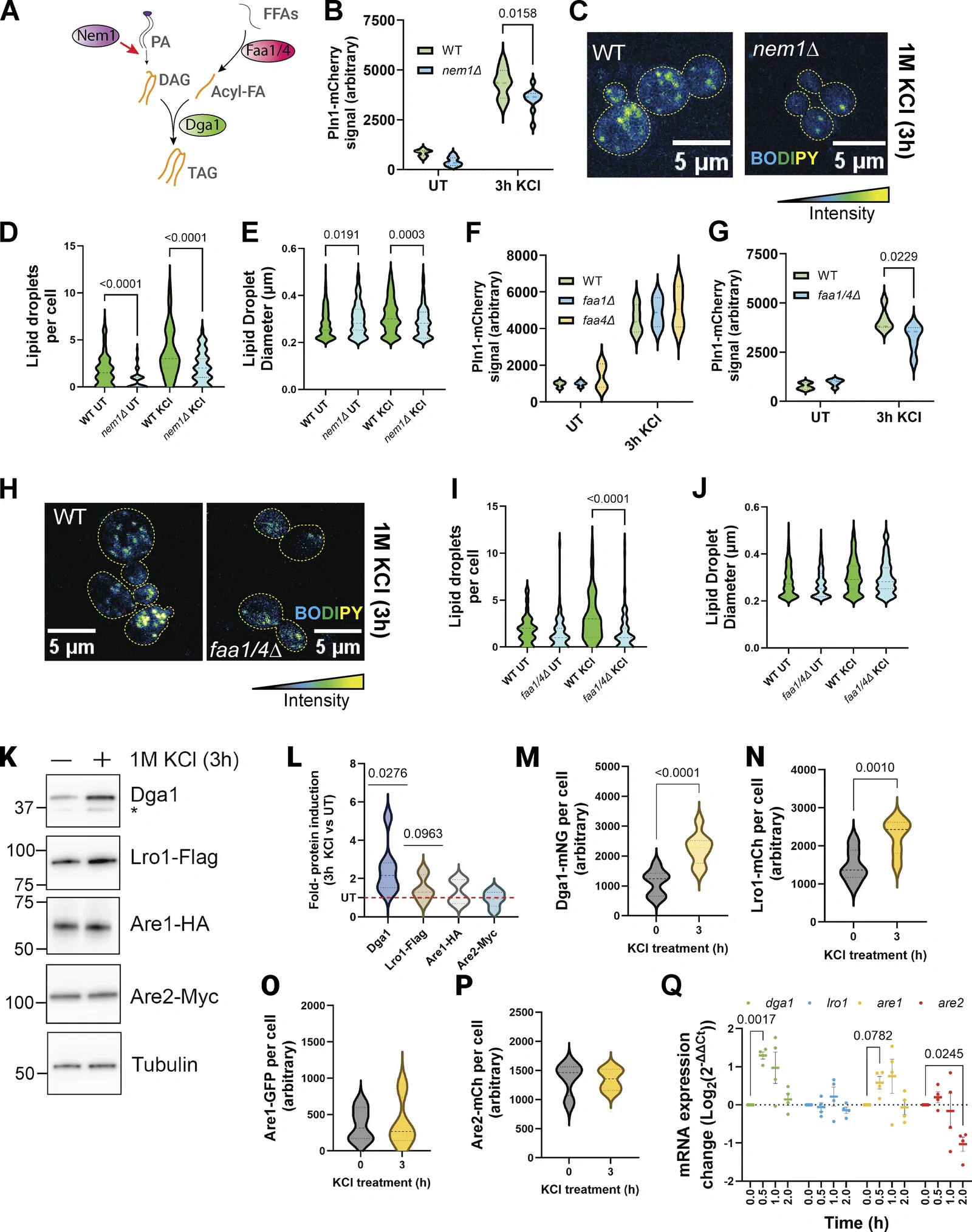
**DAG and acylated fatty acid availability limits LD accumulation. (A)** Schematic of TAG production from PA via DAG and FFAs. **(B)** Median Pln1-mCherry fluorescence measured by flow cytometry from populations of single WT and *nem1Δ* cells subjected to 1M KCl treatment in SCD media for 3 h. UT = untreated; PA, phosphatidic acid; FFAs, free fatty acids. Violin plots show the spread of data, with the dashed lines indicating the median and the dotted lines the upper and lower quartiles. *N* = 10. **(C)** LDs stained with BODIPY and imaged by live-cell microscopy after 3 h of 1M KCl treatment in SCD for Pln1-mCherry WT and *nem1Δ* cells. Z-projection images are representative of multiple replicates. **(D)** Analysis of LDs per cell from samples used in C. UT = untreated. Violin plots show the spread of data, with the dashed lines indicating the median and the dotted lines the upper and lower quartiles. *N* = 104 (WT UT), 88 (*nem1Δ* UT), 109 (WT KCl), 95 (*nem1Δ* KCl) cells from three biological replicates. **(E)** Analysis of LD diameters from samples used in C. UT = untreated. Violin plots show the spread of data, with the dashed lines indicating the median and the dotted lines the upper and lower quartiles. *N* = 267 (WT UT), 333 (*nem1Δ* UT), 199 (WT KCl), 233 (*nem1Δ* KCl) LDs from 3 biological replicates. **(F)** Median Pln1-mCherry fluorescence measured by flow cytometry from populations of WT, *faa1Δ*, and *faa4Δ* cells subjected to 1M KCl treatment in SCD media for 3 h. UT = untreated. Violin plots show the spread of data, with the dashed lines indicating the median and the dotted lines the upper and lower quartiles. *N* = 4. **(G)** Median Pln1-mCherry fluorescence measured by flow cytometry from populations of WT and *faa1Δfaa4Δ* cells subjected to 1M KCl treatment in SCD media for 3 h. UT = untreated. Violin plots show the spread of data, with the dashed lines indicating the median and the dotted lines the upper and lower quartiles. *N* = 5. **(H)** LDs stained with BODIPY and imaged by live-cell microscopy after 3 h of 1M KCl treatment in SCD for Pln1-mCherry WT and *faa1Δfaa4Δ* cells. Z-projection images are representative of multiple replicates. **(I)** Analysis of LDs per cell from samples used in H. UT = untreated. Violin plots show the spread of data, with the dashed lines indicating the median and the dotted lines the upper and lower quartiles. *N* = 89 (WT UT), 82 (*faa1Δfaa4Δ* UT), 79 (WT KCl), 96 (*faa1Δfaa4Δ* KCl) cells from three biological replicates. **(J)** Analysis of LD diameters from samples used in H. UT = untreated. Violin plots show the spread of data, with the dashed lines indicating the median and the dotted lines the upper and lower quartiles. *N* = 245 (WT UT), 267 (*faa1Δfaa4Δ* UT), 459 (WT KCl), 199 (*faa1Δfaa4Δ* KCl) LDs from three biological replicates. **(K)** Western blot analysis of Dga1, Lro1-Flag, Are1-HA, and Are2-Myc levels before and after 3 h of 1M KCl treatment in SCD. Images are representative of multiple replicates. **(L)** Analysis of normalized protein fold change from F. Statistics are compared with 1 (no fold change). UT = untreated. Violin plots show the spread of data, with the dashed lines indicating the median and the dotted lines the upper and lower quartiles. *N* = 7. **(M)** Median Dga1-mNG fluorescence measured by flow cytometry from populations of cells subjected to 1M KCl treatment in SCD media for 3 h. UT = untreated. Violin plots show the spread of data, with the dashed lines indicating the median and the dotted lines the upper and lower quartiles. *N* = 8. **(N)** Median Lro1-mCherry fluorescence measured by flow cytometry from cells subjected to 1M KCl treatment in SCD media for 3 h. UT = untreated. Violin plots show the spread of data, with the dashed lines indicating the median and the dotted lines the upper and lower quartiles. *N* = 6. **(O)** Median Are1-GFP fluorescence measured by flow cytometry from cells subjected to 1M KCl treatment in SCD media for 3 h. UT = untreated. Violin plots show the spread of data, with the dashed lines indicating the median and the dotted lines the upper and lower quartiles. *N* = 4. **(P)** Median Are2-mCherry fluorescence measured by flow cytometry from cells subjected to 1M KCl treatment in SCD media for 3 h. UT = untreated. Violin plots show the spread of data, with the dashed lines indicating the median and the dotted lines the upper and lower quartiles. *N* = 4. **(Q)** mRNA levels of *DGA1, LRO1, ARE1,* and *ARE2* compared with the housekeeping gene *TUB2* at the indicated times after 1M KCl treatment. Violin plots show the spread of data, with the dashed lines indicating the median and the dotted lines the upper and lower quartiles. *N* = 4. Source data are available for this figure:SourceData FS3.

Dga1 uses DAG and CoA-activated fatty acids to produce TAG (Fig. S3 A) (Oelkers et al., 2002). As an independent test on the important role of Dga1, we restricted the availability of its substrates. Deletion of Nem1 or Faa1 and Faa4, which reduce the levels of DAG (Su et al., 2014) and activated fatty acids (Johnson et al., 1994), respectively, impaired LD accumulation under hyperosmotic stress (Fig. S3, B–J), consistent with a key role for Dga1 in this adaptive response.

### Dga1 protein levels increase upon hyperosmotic stress

Having established the importance of Dga1 for stress-induced LD accumulation, we next examined how Dga1 is regulated during hyperosmotic stress. Hyperosmotic stress resulted in a significant increase in endogenous Dga1 protein abundance, as detected by western blot and by flow cytometry of endogenously tagged Dga1 (Fig. S3, K–M). Importantly, this effect appeared specific as levels of all other neutral lipid–synthesizing enzymes remained unchanged aside from a modest increase in the levels of endogenous Lro1 expressed as a fusion to mCherry (Fig. S3, K–P). Consistent with the increase in protein abundance, *DGA1* mRNA levels were also specifically increased (Fig. S3 Q).

We next used fluorescence microscopy of Dga1-mNeonGreen (Dga1-mNG) to determine whether the increase in Dga1 abundance was accompanied by changes in subcellular localization. In unstressed cells, Dga1-mNG was distributed throughout the ER, whereas under hyperosmotic stress, it concentrated at LDs (Fig. 2 F). Given that Dga1 is more long-lived on LDs than in the ER (Ruggiano et al., 2016), this relocalization likely further stabilizes Dga1. Together, these data indicate that Dga1 upregulation and relocalization drive LD accumulation during adaptation to hyperosmotic stress.

### The canonical hyperosmotic stress response indirectly limits LD accumulation

**Figure S4.**
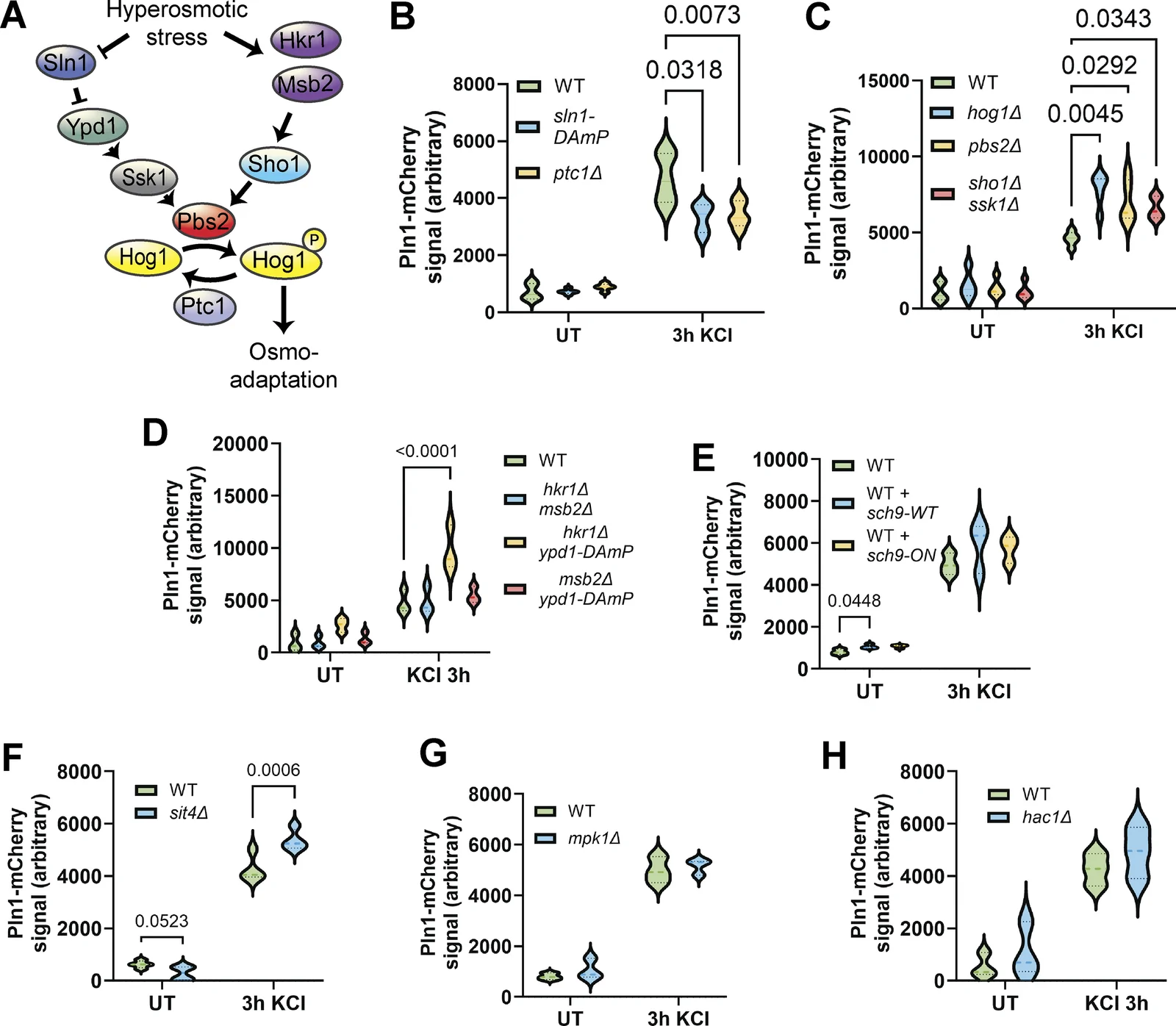
**Hog1 regulator effect on LD accumulation upon hyperosmotic stress. (A)** Schematic of the Hog1 regulatory pathway. **(B)** Median Pln1-mCherry fluorescence measured by flow cytometry from populations of single WT, *sln1-DAmP*, and *ptc1Δ* cells subjected to 1M KCl treatment in SCD media for 3 h. UT = untreated. Ptc1 is the phosphatase which turns off Hog1 activity, while Sln1 constrains the activity of the signaling cascade upstream of Hog1. Violin plots show the spread of data, with the dashed lines indicating the median and the dotted lines the upper and lower quartiles. *N* = 5. **(C)** Median Pln1-mCherry fluorescence measured by flow cytometry from populations of single WT, *hog1Δ*, *pbs2Δ*, and *sho1Δssk1Δ* cells subjected to 1M KCl treatment in SCD media for 3 h. UT = untreated. Pbs2 is the MAP kinase kinase which activates Hog1, while Sho1 and Ssk1 are the MAP kinase kinase kinases which activated Pbs2. These mutations restrict Hog1 activity. Violin plots show the spread of data, with the dashed lines indicating the median and the dotted lines the upper and lower quartiles. *N* = 5. **(D)** Median Pln1-mCherry fluorescence measured by flow cytometry from populations of single WT, *hkr1Δmsb2Δ*, *hkr1Δypd1-DAmP*, and *msb2Δypd1-DAmP* cells subjected to 1M KCl treatment in SCD media for 3 h. UT = untreated. These strains each have mutations of the most upstream sensor proteins, restricting activation of the HOG pathway. Violin plots show the spread of data, with the dashed lines indicating the median and the dotted lines the upper and lower quartiles. *N* = 3. **(E)** Median Pln1-mCherry fluorescence measured by flow cytometry from populations of single WT cells with no vector, Sch9-3xHA vector, or Sch9-2D3E-3xHA vector (Sch9 ON) subjected to 1M KCl treatment in SCD media for 3 h. UT = untreated. Expressing the Sch9-ON vector prevents the inhibition of Sch9 signaling following TORC1 inhibition. Violin plots show the spread of data, with the dashed lines indicating the median and the dotted lines the upper and lower quartiles. *N* = 3. **(F)** Median Pln1-mCherry fluorescence measured by flow cytometry from populations of single WT and *sit4Δ* cells subjected to 1M KCl treatment in SCD media for 3 h. UT = untreated. Sit4 is activated upon TORC1 inhibition, and its deletion prevents this activation. Violin plots show the spread of data, with the dashed lines indicating the median and the dotted lines the upper and lower quartiles. *N* = 4. **(G)** Median Pln1-mCherry fluorescence measured by flow cytometry from populations of single WT and *mpk1Δ* cells subjected to 1M KCl treatment in SCD media for 3 h. UT = untreated. Mpk1 is the terminally activated kinase in the cell wall integrity pathway, and its deletion prevents this activation. Violin plots show the spread of data, with the dashed lines indicating the median and the dotted lines the upper and lower quartiles. *N* = 3. **(H)** Median Pln1-mCherry fluorescence measured by flow cytometry from populations of single WT and *hac1Δ* cells subjected to 1M KCl treatment in SCD media for 3 h. UT = untreated. *HAC1* mRNA undergoes splicing upon UPR activation, with the resulting protein activating transcription of downstream targets. Gene deletion prevents this occurring. Violin plots show the spread of data, with the dashed lines indicating the median and the dotted lines the upper and lower quartiles. *N* = 3. UPR, unfolded protein response.

**Figure 3.**
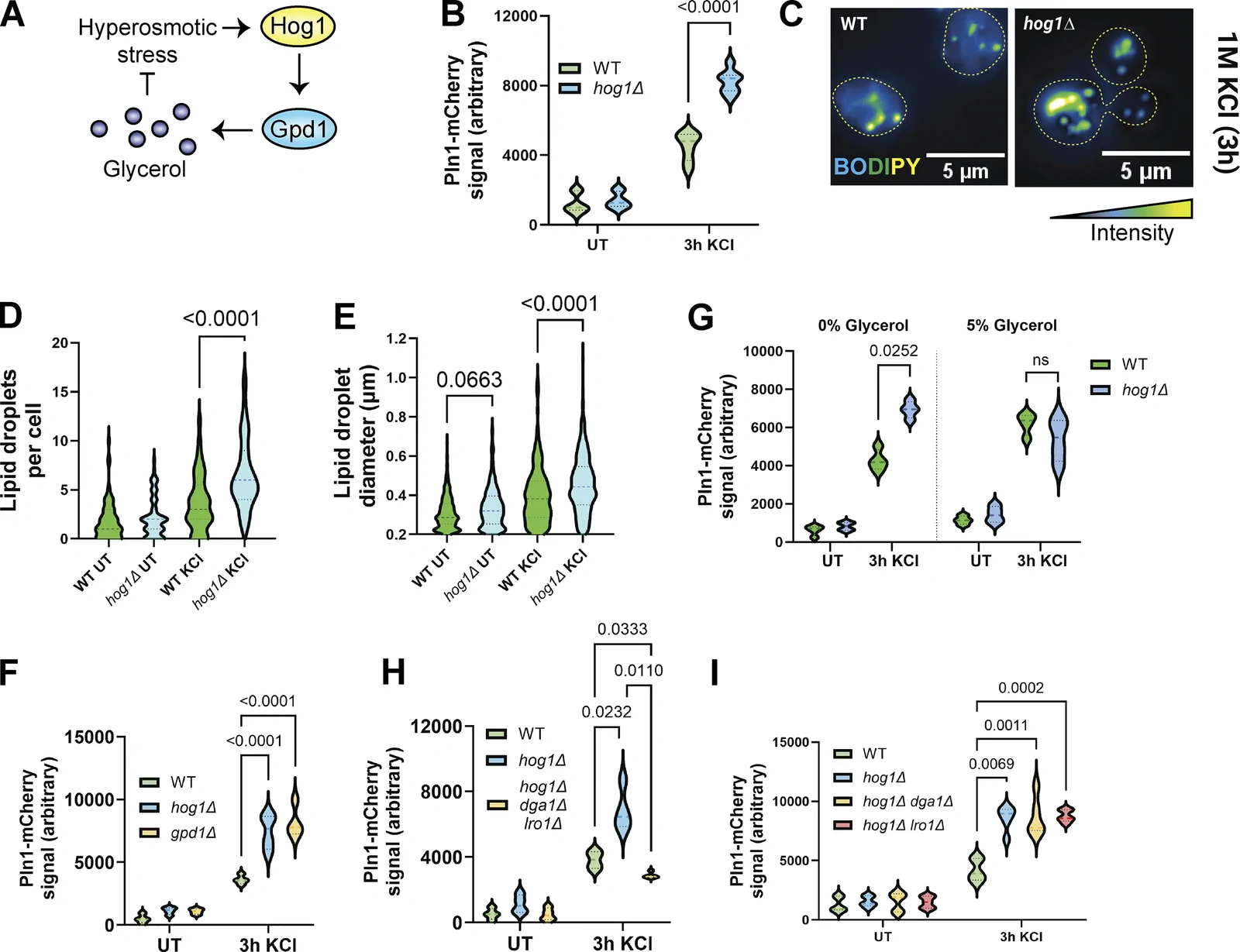
**Canonical hyperosmotic stress signaling indirectly limits LD production. (A)** Simplified schematic of hyperosmotic stress adaptation signaling. Hyperosmotic stress leads to activation of the Hog1 MAP kinase, which enhances Gpd1 production to create glycerol as a balancing intracellular osmolyte. **(B)** Median Pln1-mCherry fluorescence measured by flow cytometry from populations of single WT and *hog1Δ* cells subjected to 1M KCl treatment in SCD media for 3 h. UT = untreated. Violin plots show the spread of data, with the dashed lines indicating the median and the dotted lines the upper and lower quartiles. *N* = 7. **(C)** LDs stained with BODIPY and imaged by live-cell microscopy after 3 h of 1M KCl treatment in SCD for Pln1-mCherry WT and *hog1Δ* cells. Z-projection images are representative of multiple replicates. **(D)** Analysis of LDs per cell from samples used in C. UT = untreated. Violin plots show the spread of data, with the dashed lines indicating the median and the dotted lines the upper and lower quartiles. *N* = 83 (WT UT), 83 (*hog1Δ* UT), 49 (WT KCl), 55 (*hog1Δ* KCl) cells from three biological replicates. **(E)** Analysis of LD diameters from samples used in C. UT = untreated. Violin plots show the spread of data, with the dashed lines indicating the median and the dotted lines the upper and lower quartiles. *N* = 293 (WT UT), 238 (*hog1Δ* UT), 188 (WT KCl), 396 (*hog1Δ* KCl) LDs from three biological replicates. **(F)** Median Pln1-mCherry fluorescence measured by flow cytometry from populations of single WT, *hog1Δ*, and *gpd1Δ* cells subjected to 1M KCl treatment in SCD media for 3 h. UT = untreated. Violin plots show the spread of data, with the dashed lines indicating the median and the dotted lines the upper and lower quartiles. *N* = 4. **(G)** Median Pln1-mCherry fluorescence measured by flow cytometry from populations of single WT and *hog1Δ* cells subjected to 1M KCl treatment with or without 5% glycerol in SCD media for 3 h. UT = untreated. Violin plots show the spread of data, with the dashed lines indicating the median and the dotted lines the upper and lower quartiles. *N* = 4. **(H)** Median Pln1-mCherry fluorescence measured by flow cytometry from populations of single WT, *hog1Δ*, and *hog1Δdga1Δlro1Δ* cells subjected to 1M KCl treatment in SCD media for 3 h. UT = untreated. Violin plots show the spread of data, with the dashed lines indicating the median and the dotted lines the upper and lower quartiles. *N* = 4. **(I)** Median Pln1-mCherry fluorescence measured by flow cytometry from populations of single WT, *hog1Δ*, *hog1Δdga1Δ*, and *hog1Δlro1Δ* cells subjected to 1M KCl treatment in SCD media for 3 h. UT = untreated. Violin plots show the spread of data, with the dashed lines indicating the median and the dotted lines the upper and lower quartiles. *N* = 5.

Hyperosmotic stress adaptation is predominantly mediated through the high osmolarity glycerol (HOG) pathway (de Nadal and Posas, 2022). This well-characterized signaling cascade involves multiple sensors that converge on activation of the Hog1 MAP kinase (Fig. S4 A), which triggers an adaptive transcriptional program. Among other changes, this program rapidly enhances synthesis of glycerol, which acts as an intracellular osmolyte and facilitates restoration of cell volume (Fig. 3 A). Hog1 activation typically lasts for around 30 min, while downstream protein and glycerol synthesis peak within 60–90 min of stress onset (Petelenz-Kurdziel et al., 2013; Shen et al., 2023; Klipp et al., 2005). To test whether the LD accumulation upon hyperosmotic stress is under HOG pathway control, we analyzed *hog1Δ* cells. In the absence of stress, *hog1Δ* cells showed Pln1-mCherry levels comparable to WT controls (Fig. 3 B). Remarkably, under hyperosmotic stress, *hog1Δ* cells displayed a dramatic increase in Pln1-mCherry levels, far exceeding that observed in WT cells (Fig. 3 B). This pronounced Pln1-mCherry increase in *hog1Δ* cells reflected the accumulation of more and larger LDs, as confirmed by fluorescence microscopy of BODIPY-stained cells (Fig. 3, C–E). Moreover, manipulation of Hog1 activity by mutations that promote or inhibit its function resulted in a reduction or increase in Pln1-mCherry accumulation, respectively (Fig. S4, B and C). Together, these experiments indicate that the HOG pathway limits LD accumulation during hyperosmotic stress.

The HOG pathway could limit LD accumulation because when it is active, cells ultimately experience less of the hyperosmotic stress. In this scenario, one would expect that LD accumulation should be similarly modulated by altering glycerol levels. Indeed, deletion of *GPD1*, a glycerol phosphate dehydrogenase essential for glycerol production, enhanced Pln1-mCherry accumulation to the same extent as *HOG1* deletion (Fig. 3 F). Conversely, supplementing *hog1Δ* cells with glycerol reduced Pln1-mCherry accumulation to WT levels (Fig. 3 G). Blocking activation of the upstream sensor proteins Hkr1, Msb2, and Ypd1 did not prevent LD accumulation (Fig. S4 D), further indicating that LD accumulation is not directly regulated by the HOG pathway. As cells defective in the HOG pathway cannot effectively produce the intracellular osmolytes necessary to resolve the decreased membrane tension upon acute hyperosmotic stress (Petelenz-Kurdziel et al., 2013), LDs may be continually produced to try and increase membrane tension in these cells, as described for the acute phase of hyperosmotic stress (Phan et al., 2025). In agreement with this, although *hog1Δ* cells relied on Dga1 and Lro1 for full LD accumulation (Fig. 3 H), this was no longer dependent upon Dga1 as in the WT (Fig. 3 I and Fig. 2 B). This suggests a larger role for Lro1, as seen in the acute phase of adaptation (Phan et al., 2025). Together, these results indicate that the HOG pathway indirectly limits LD accumulation by promoting cell adaptation to hyperosmotic stress.

Besides the HOG pathway, the response to hyperosmotic stress can also be modulated by a variety of other signaling pathways such as the unfolded protein response (Burgos et al., 2019), the cell wall integrity (Yoshikawa et al., 2009), and target of rapamycin (Hughes Hallett et al., 2014). However, genetic manipulation of these pathways did not affect Pln1-mCherry upon hyperosmotic stress levels, suggesting they play no role in the LD response (Fig. S4, E–H).

### Chronic hyperosmotic stress drives membrane remodeling

**Figure 4.**
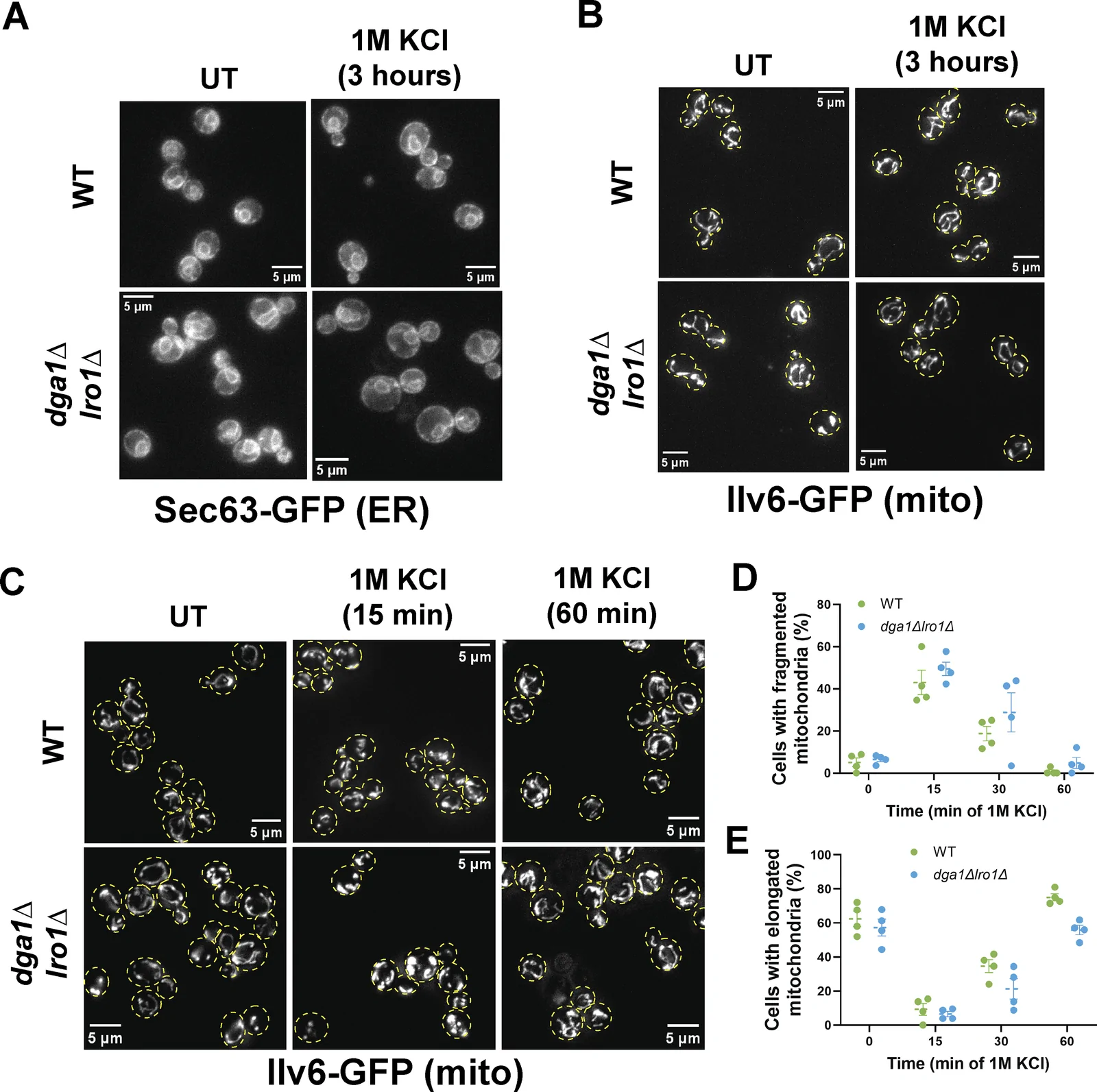
**Hyperosmotic stress does not cause lasting changes to ER or mitochondrial morphology. (A)** Cellular ER visualized with Sec63-GFP and imaged by live-cell microscopy after 0 (UT) or 3 h of 1M KCl treatment in SCD for WT and *dga1Δlro1Δ* cells. Images show cell midsections and are representative of multiple replicates. **(B)** Mitochondria visualized by endogenous Ilv6-GFP expression and imaged by live-cell microscopy after 0 (UT) or 3 h of 1M KCl treatment in SCD for WT and *dga1Δlro1Δ* cells. Z-projection images are representative of multiple replicates. **(C)** Mitochondria visualized by endogenous Ilv6-GFP expression and imaged by live-cell microscopy after 0 (UT), 15, or 60 min of 1M KCl treatment in SCD for WT and *dga1Δlro1Δ* cells. Z-projection images are representative of multiple replicates. **(D)** Quantification of cells from C with fragmented mitochondria. Each point shows the percentage of cells from a replicate (*N* = 4) exhibiting solely fragmented mitochondria. Error bars show the mean and SEM. **(E)** Quantification of cells from C with elongated mitochondria. Each point shows the percentage of cells from a replicate (*N* = 4) exhibiting solely elongated mitochondria. Error bars show the mean and SEM.

While our data identify TAG-containing LDs as important for adaptation to hyperosmotic stress, we next asked whether this response is accompanied by broader organelle remodeling. To assess how widespread organelle remodeling is during hyperosmotic stress, we analyzed the morphology of the ER and mitochondria, organelles that maintain close physical and metabolic connections with LDs (Wu et al., 2018; Henne and Cohen, 2026). Upon hyperosmotic stress, we detected no substantial changes in the morphology or abundance of the ER and mitochondria, monitored, respectively, by endogenously GFP-tagged Sec63 and Ilv6 (Fig. 4, A and B), well-established marker proteins of these organelles (Wang et al., 2018; Huh et al., 2003). Time course analysis revealed that as for other stress conditions (Fedoseeva et al., 2017; Kondo et al., 2022), hyperosmotic stress caused a rapid but transient mitochondrial fragmentation. This was resolved within 1 h in both WT and *dga1Δlro1Δ* cells (Fig. 4, C–E). Thus, although hyperosmotic stress causes transient mitochondrial fragmentation during the acute phase, chronic hyperosmotic stress does not trigger persistent large-scale organelle remodeling. LD accumulation therefore appears to be a specific long-term lipid response rather than a consequence of gross ER or mitochondrial dysfunction. Importantly, the similar organelle morphology observed in WT and *dga1Δlro1Δ* cells further indicates that the reduced fitness of TAG-deficient cells under chronic hyperosmotic stress is not caused by gross ER or mitochondrial collapse.

**Figure S5.**
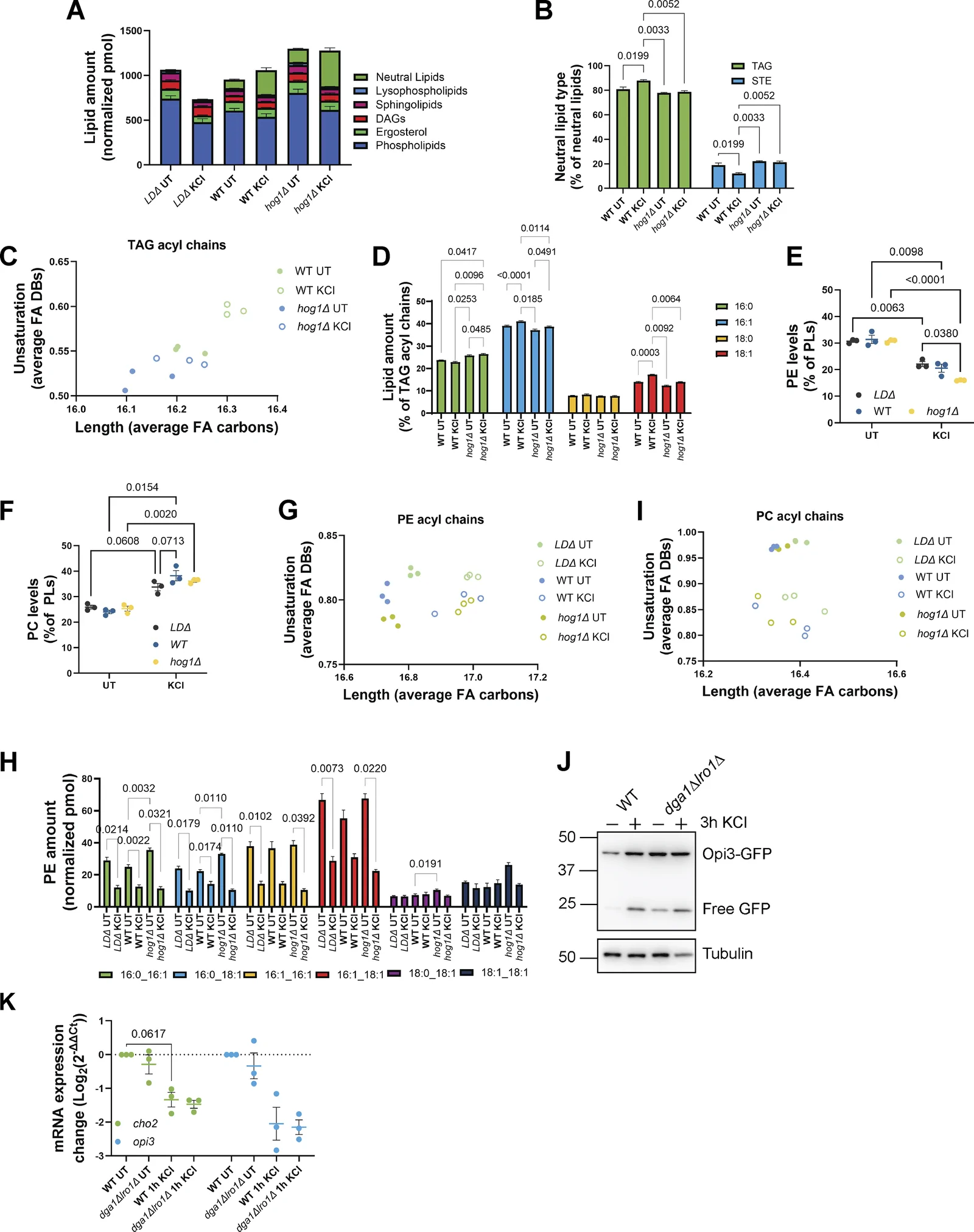
**Limiting PC catabolism does not impact LDs under hyperosmotic stress. (A)** Normalized levels of the major lipid classes for each strain detected by lipidomics with and without 3 h of treatment with 1M KCl. Bars show the mean with SEM from three biological replicates. **(B)** Proportion of neutral lipids from A that were TAGs and STEs for WT and *hog1Δ* cells with and without 3 h of treatment with 1M KCl. Bars show the mean with SEM from three biological replicates. **(C)** Averaged properties of all acyl chains found in TAGs for WT and *hog1Δ* cells both with and without 3 h of 1M KCl treatment. The x axis shows the average length in carbons, while the y axis shows the average number of double bonds. Each point shows an individual replicate (three for each condition). **(D)** Proportion of the major acyl-chain classes found in TAGs for WT and *hog1Δ* cells with and without 3 h of treatment with 1M KCl. Bars show the mean with SEM from three biological replicates. **(E)** PE species as a percentage of total PLs detected with and without 3 h of treatment with 1M KCl. *N* = 3; error bars show the mean and SEM. **(F)** PC species as a percentage of total PLs detected with and without 3 h of treatment with 1M KCl. *N* = 3; error bars show the mean and SEM. **(G)** Averaged properties of all acyl chains found in PE for each strain both with and without 3 h of 1M KCl treatment. The x axis shows the average length in carbons, while the y axis shows the average number of double bonds. Each point shows an individual replicate (three for each condition). **(H)** Normalized levels of the major classes of PE species for each strain with and without 3 h of treatment with 1M KCl. Bars show the mean with SEM from three biological replicates. **(I)** Averaged properties of all acyl chains found in PC for each strain both with and without 3 h of 1M KCl treatment. The x axis shows the average length in carbons, while the y axis shows the average number of double bonds. Each point shows an individual replicate (three for each condition). **(J)** Western blot of Opi3-GFP from populations of single WT and *dga1Δlro1Δ* cells either untreated or subjected to 1M KCl treatment in SCD media for 3 h. Images are representative of multiple replicates. **(K)** mRNA levels of *CHO2* and *OPI3* in WT and *dga1Δlro1Δ* cells compared with the housekeeping gene *TUB2* at the indicated times after 1M KCl treatment. Error bars show the mean and SEM, with four replicates for *CHO2* and 3 for *OPI3*. PLs, phospholipids. Source data are available for this figure: SourceData FS5.

**Figure 5.**
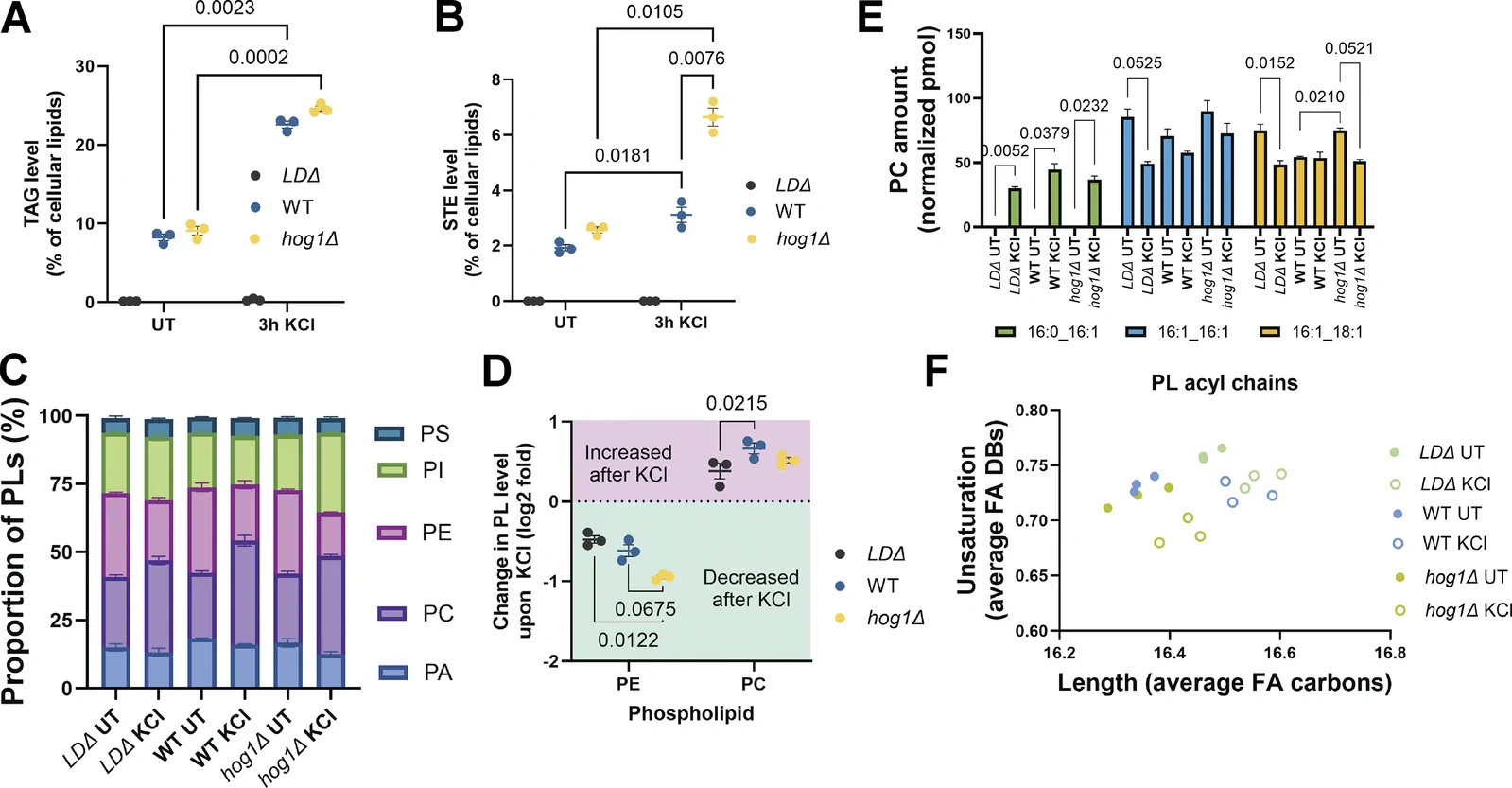
**Chronic hyperosmotic stress drives LD-dependent lipidome changes. (A)** TAGs as a percentage of total cellular lipids detected by lipidomics with and without 3 h of treatment with 1M KCl. *N* = 3; error bars show the mean and SEM. **(B)** STEs as a percentage of total cellular lipids detected by lipidomics with and without 3 h of treatment with 1M KCl. *N* = 3; error bars show the mean and SEM. **(C)** Proportion of phospholipid species detected by lipidomics as a percentage of total PLs. PA = phosphatidic acid; PC = phosphatidylcholine; PE = phosphatidylethanolamine; PI = phosphatidylinositol; PS = phosphatidylserine; PLs, phospholipids. *N* = 3; error bars show the mean and SEM. **(D)** Log2 fold changes in PE and PC detected by lipidomics following 3 h of treatment with 1M KCl compared with untreated. *N* = 3; error bars show the mean and SEM. **(E)** Normalized levels of the major classes of PC species detected by lipidomics for each strain with and without 3 h of treatment with 1M KCl. *N* = 3; error bars show the mean and SEM. **(F)** Averaged properties of all acyl chains found in phospholipids for each strain both with and without 3 h of 1M KCl treatment. The x axis shows the average length in carbons, while the y axis shows the average number of double bonds. Each point shows an individual replicate (three for each condition).

Given the absence of widespread organelle remodeling, we next asked whether hyperosmotic stress instead remodels the cellular lipidome. To address this, we performed whole-cell lipidomic analysis to define global lipid changes. We observed that hyperosmotic stress resulted in a large increase of neutral lipids in both WT and *hog1Δ* cells but not in *LDΔ*, as expected (Fig. S5 A). Interestingly, in WT cells this increase was dominated by TAGs, while in *hog1Δ* cells, TAGs and STEs increased to a similar extent suggesting that the LDs produced in these different genetic backgrounds are distinct (Fig. 5, A and B; and Fig. S5 B). Further analysis of the TAGs accumulated during hyperosmotic stress revealed changes in the acyl-chain composition specific to WT cells, with the constituent fatty acids being, on average, longer and more unsaturated (Fig. S5 C). These changes were primarily due to higher proportions of 16:1 and 18:1 chains (Fig. S5 D). Thus, chronic hyperosmotic stress remodels neutral lipid content and composition.

We next examined changes to phospholipid composition. Most phospholipids remained largely unchanged as a proportion of the phospholipid pool (Fig. 5 C). Strikingly, however, in all three strains hyperosmotic stress increased the proportion of PC with a concomitant drop in phosphatidylethanolamine (PE) (Fig. 5 D; and Fig. S5, E and F). The decrease in PE was comparable between WT and *LD*Δ cells but more pronounced in *hog1*Δ cells, whereas the increase in PC was attenuated in *LD*Δ cells relative to WT and trended slightly lower in *hog1*Δ (Fig. 5 D). Hyperosmotic stress therefore drives a switch in the major phospholipid classes, a change that appears more robust in WT cells. We then asked whether this switch was accompanied by remodeling of the acyl chains, both within each class and in the phospholipids as a whole. The PE acyl chains became longer following stress in all strains, owing to a depletion of C16-containing species, while the minor di-C18 species were retained (Fig. S5, G and H). PC chains remained similar in length but became more saturated, driven by the emergence of a new species, 16:0_16:1, absent from untreated cells (Fig. 5 E and Fig. S5 I). The overall acyl-chain profile of stressed WT cells shifted toward longer chains but remained closely similar to that of *LD*Δ cells (Fig. 5 F); because *LD*Δ cells share this profile yet are impaired for proliferation under stress, acyl-chain remodeling alone cannot account for the fitness defect. Thus, chronic hyperosmotic stress remodels the most abundant phospholipids, shifting the balance from PE to PC and altering the acyl-chain composition within both classes.

### Interplay between LDs and phospholipid remodeling during chronic hyperosmotic stress

**Figure 6.**
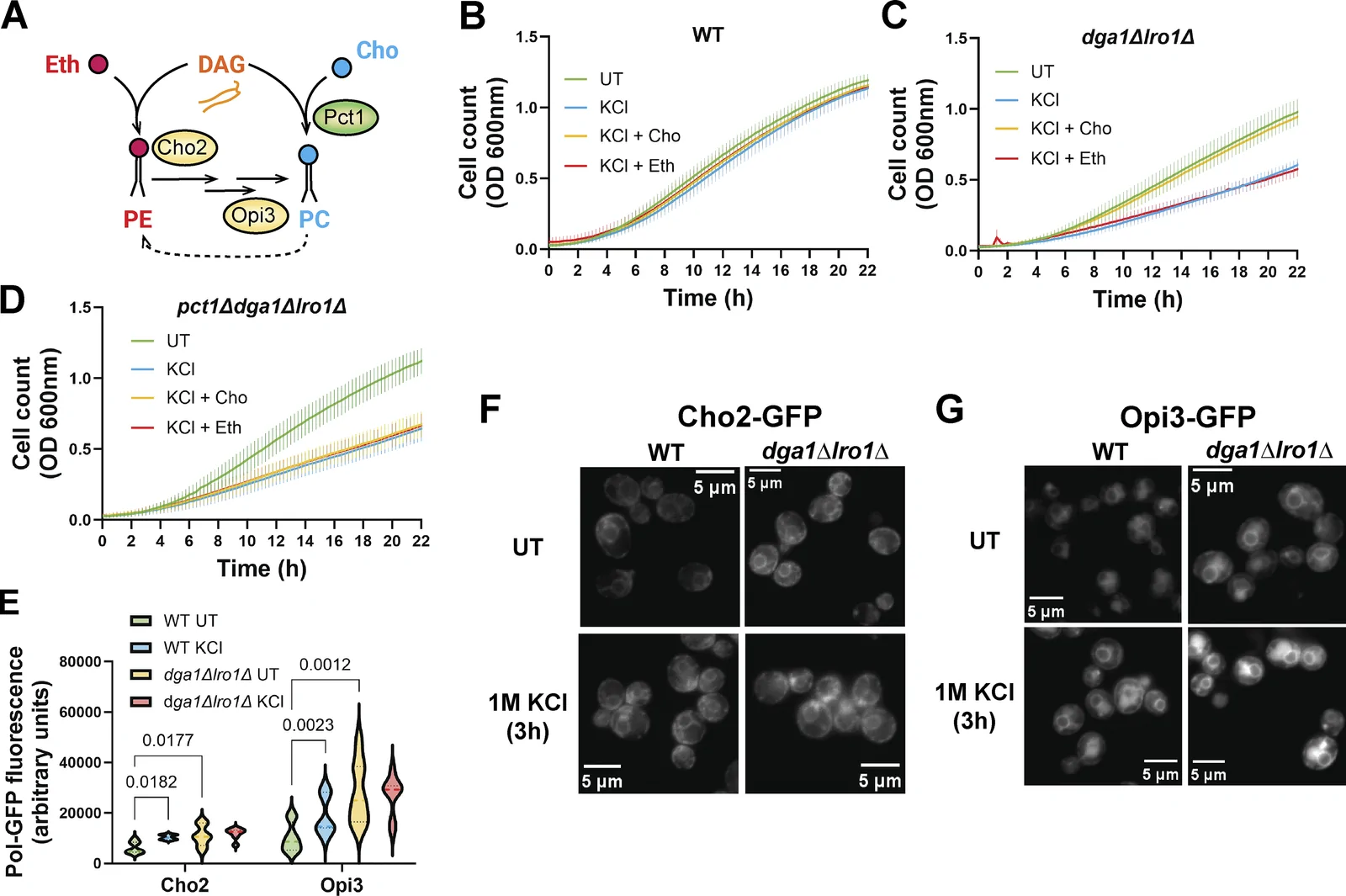
**PE and PC must be balanced for hyperosmotic stress adaptation. (A)** Schematic of PC biosynthesis from PE conversion. PE and PC can also be made by combining DAG with, respectively, ethanolamine (Eth) or choline (Cho). **(B)** Growth assay of Pln1-mCherry WT cells in SCD and SCD + 1M KCl with and without supplementation of 2 mM ethanolamine (Eth) or choline (Cho). The main line shows the mean, with the error bars showing SEM from six biological replicates. **(C)** Growth assay of Pln1-mCherry *dga1Δlro1Δ* cells in SCD and SCD + 1M KCl with and without supplementation of 2 mM ethanolamine (Eth) or choline (Cho). The main line shows the mean, with the error bars showing SEM from six biological replicates. **(D)** Growth assay of Pln1-mCherry *dga1Δlro1Δpct1Δ* cells in SCD and SCD + 1M KCl with and without supplementation of 2 mM ethanolamine (Eth) or choline (Cho). The main line shows the mean, with the error bars showing SEM from six biological replicates. **(E)** Median fluorescence of endogenously expressed Cho2-GFP and Opi3-GFP measured by flow cytometry from populations of WT and *dga1Δlro1Δ* cells subjected to 1M KCl treatment in SCD media for 3 h. UT = untreated. Violin plots show the spread of data, with the dashed lines indicating the median and the dotted lines the upper and lower quartiles. *N* = 7 for Cho2-GFP, 8 for Opi3-GFP. **(F)** Localization of Cho2 tagged with GFP in WT and *dga1Δlro1Δ* cells grown either in SCD media or in SCD supplemented with 1M KCl for 3 h prior to imaging. Images use the same color scale, show a single slice, and are representative of multiple experiments. **(G)** Localization of Opi3 tagged with GFP in WT and *dga1Δlro1Δ* cells grown either in SCD media or in SCD supplemented with 1M KCl for 3 h prior to imaging. Images use the same color scale, show a single slice, and are representative of multiple experiments.

To test whether the increased PC is functionally important, we manipulated PC synthesis. PC is produced by sequential trimethylation of PE in the ER, with the initial step carried out by Cho2 and subsequent steps by Opi3 (McMaster and Bell, 1994) (Fig. 6 A). Alternatively, PC can be synthesized via the Kennedy pathway from DAG and choline, with Pct1 catalyzing the rate-limiting step (Fig. 6 A). The Kennedy pathway can also produce PE by providing ethanolamine instead of choline. We therefore asked whether stimulating PC synthesis could compensate for the loss of TAG in *dga1Δlro1*Δ cells. Choline supplementation improved the growth of *dga1Δlro1Δ* cells under hyperosmotic stress (Fig. 6, B and C). Importantly, this effect was dependent on Pct1, indicating that the rescue requires PC synthesis from exogenous choline (Fig. 6 D). In contrast, ethanolamine supplementation did not improve the growth of *dga1Δlro1Δ* cells under hyperosmotic stress. Thus, elevated PC and TAG levels are both important for adaptation to chronic hyperosmotic stress, and increasing PC levels can compensate for the absence of TAG.

Having established that elevated PC contributes to cell fitness under hyperosmotic stress, we next investigated how the PC increase is triggered. We hypothesized that the key PC biosynthetic enzymes Cho2 and Opi3 might be upregulated upon hyperosmotic stress. Quantification of endogenously GFP-tagged Cho2 (Cho2-GFP) and Opi3 (Opi3-GFP) by flow cytometry showed that chronic hyperosmotic stress increases the levels of both enzymes (Fig. 6 E). This increase did not result in altered localization of either Cho2-GFP or Opi3-GFP (Fig. 6, F and G) although in Opi3-GFP–expressing cells some of the GFP signal could be found within the vacuole. Using western blot, we confirmed both that this vacuolar species was likely free GFP released by autophagy and that the full-length Opi3-GFP was increased following hyperosmotic stress (Fig. S5 J). Surprisingly, in both cases, the increase in protein occurred without a corresponding increase in mRNA (Fig. S5 K), indicating a posttranscriptional mechanism. Increased Cho2 and Opi3 levels following hyperosmotic stress likely enable the phospholipid shift from PE to PC.

To further explore the links between TAG and PC synthesis in response to hyperosmotic stress, we tested how blocking TAG production impacts PC synthesis. Surprisingly, *dga1Δlro1Δ* cells displayed higher steady-state levels of both Cho2-GFP and Opi3-GFP, which did not increase further following hyperosmotic stress, and were not mislocalized (Fig. 6, E–G and Fig. S5 J). This increase also occurred without a corresponding increase in mRNA (Fig. S5 K). Thus, in the absence of TAG synthesis, PC biosynthetic enzymes are constitutively elevated and already at, or close to, the levels reached by stressed WT cells. This may explain why choline supplementation rescues growth in *dga1Δlro1Δ* cells: with the enzymes already maximally induced, these cells cannot further increase PC synthesis in response to stress, a limitation that is bypassed by supplying exogenous choline (Fig. 6 C).

### Membrane adaptation to hyperosmotic stress requires TAG synthesis

**Figure 7.**
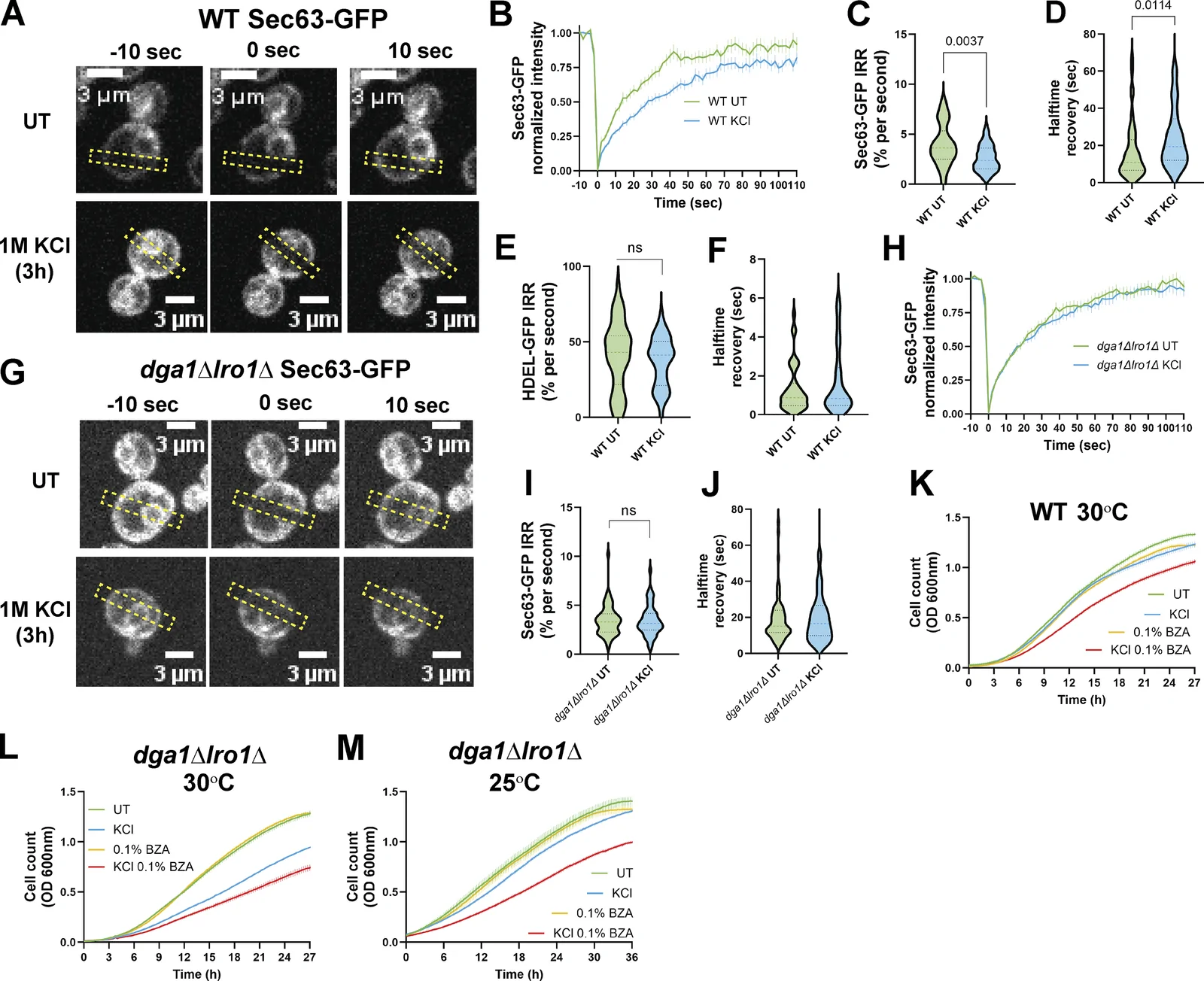
**Membrane adaptation to hyperosmotic stress requires TAG synthesis. (A)** Representative image of endogenously expressing Sec63-GFP WT cell midsections subjected to FRAP. Cells were subject to 0 or 3 h of treatment with 1M KCl in SCD medium. **(B)** Normalized fluorescence recovery curves of regions of interest from A. The main line shows the mean, with the error bars showing SEM from 48 (UT) and 54 (KCl) regions subject to FRAP from three biological replicates. **(C)** IRR calculated from B. This parameter is independent of the mobile fraction. Violin plots show the spread of data, with the dashed lines indicating the median and the dotted lines the upper and lower quartiles. *N* = 48 (UT) and 54 (KCl) regions of interest from three biological replicates. **(D)** Half-time recovery (T_1/2_) calculated from B. This parameter takes into account the mobile fraction. Violin plots show the spread of data, with the dashed lines indicating the median and the dotted lines the upper and lower quartiles. *N* = 45 (UT) and 52 (KCl) regions of interest from three biological replicates. **(E)** IRR for the luminal ER substrate HDEL-GFP. This parameter is independent of the mobile fraction. Violin plots show the spread of data, with the dashed lines indicating the median and the dotted lines the upper and lower quartiles. *N* = 43 (UT) and 40 (KCl) regions of interest from three biological replicates. **(F)** Half-time recovery (T_1/2_) for the luminal ER substrate HDEL-GFP. This parameter takes into account the mobile fraction. Violin plots show the spread of data, with the dashed lines indicating the median and the dotted lines the upper and lower quartiles. *N* = 39 (UT) and 39 (KCl) regions of interest from three biological replicates. **(G)** Representative image of endogenously expressing Sec63-GFP *dga1Δlro1Δ* cell midsections subjected to FRAP. Cells were subject to 0 or 3 h of treatment with 1M KCl in SCD medium. **(H)** Normalized fluorescence recovery curves of regions of interest from G. The main line shows the mean, with the error bars showing SEM from 59 (UT) and 55 (KCl) regions subject to FRAP from three biological replicates. **(I)** IRR calculated from H. This parameter is independent of the mobile fraction. Violin plots show the spread of data, with the dashed lines indicating the median and the dotted lines the upper and lower quartiles. *N* = 59 (UT) and 55 (KCl) regions of interest from three biological replicates. **(J)** Half-time recovery (T_1/2_) calculated from H. This parameter takes into account the mobile fraction. Violin plots show the spread of data, with the dashed lines indicating the median and the dotted lines the upper and lower quartiles. *N* = 59 (UT) and 55 (KCl) regions of interest from three biological replicates. **(K)** Growth assay of Pln1-mCherry WT cells at 30^°^C in SCD and SCD + 1M KCl with and without supplementation of 0.1% BZA. The main line shows the mean, with the error bars showing SEM from 4 biological replicates. **(L)** Growth assay of Pln1-mCherry *dga1Δlro1Δ* cells at 30^ο^C in SCD and SCD + 1M KCl with and without supplementation of 0.1% BZA. The main line shows the mean, with the error bars showing SEM from four biological replicates. **(M)** Growth assay of Pln1-mCherry *dga1Δlro1Δ* cells at 25°C in SCD and SCD + 1M KCl with and without supplementation of 0.1% BZA. The main line shows the mean, with the error bars showing SEM from four biological replicates. IRR, initial recovery rates.

Next, we tested whether the lipid remodeling induced by chronic hyperosmotic stress alters membrane properties. In particular, we focused on membrane fluidity, which has been reported to decrease under hyperosmotic conditions (Laroche et al., 2001). To investigate this, we used fluorescence recovery after photobleaching (FRAP) of the ER-resident membrane protein Sec63-GFP as a readout of ER membrane protein mobility. Consistent with the previous study, Sec63-GFP diffusion in WT cells decreased following hyperosmotic stress (Fig. 7, A–D). Importantly, protein mobility within the ER lumen, detected by the HDEL-GFP reporter, was unaffected showing that the change was specific to the ER membrane (Fig. 7, E and F). Strikingly, this decrease in Sec63-GFP mobility did not occur in *dga1Δlro1Δ* cells (Fig. 7, G–J). Thus, TAG synthesis is required for the reduction in membrane protein mobility, and by inference membrane fluidity, that accompanies hyperosmotic stress.

Finally, we assessed the physiological significance of these changes in membrane properties. If reduced membrane fluidity contributes to adaptation, then artificially increasing membrane fluidity should impair growth. We therefore treated cells with benzyl alcohol (BZA), which increases membrane fluidity by intercalating into lipid bilayers and disrupting lipid packing (Mukhopadhyay et al., 2002). BZA sensitized both WT and *dga1Δlro1Δ* cells to hyperosmotic stress but had little effect in its absence (Fig. 7, K and L), demonstrating the importance of membrane homeostasis for growth under these conditions. Conversely, lowering the temperature reduces membrane fluidity (Renne and Ernst, 2023) and should alleviate the growth defect of *dga1Δlro1Δ* cells. Consistent with this prediction, reducing the growth temperature largely abolished the growth defect of *dga1Δlro1Δ* cells under hyperosmotic stress (Fig. 7 M). These results are consistent with decreased membrane fluidity being an important adaptive feature toward hyperosmotic stress, which is curtailed in the absence of TAG synthesis, reducing cell fitness.

## Discussion

We showed that chronic hyperosmotic stress triggers sustained and adaptive LD accumulation. Yeast cells frequently encounter sustained hyperosmotic environments in nature, for example, on desiccating fruit substrates and in industrial fermentations such as brewing, where high sugar concentrations impose prolonged osmotic stress. Unlike the rapid and transient LD response observed during acute osmotic shock (Phan et al., 2025), chronic stress induces persistent LD accumulation that depends predominantly on Dga1-mediated TAG synthesis and is required for optimal growth. LD accumulation is not directly driven by HOG signaling, but instead becomes more prominent when osmotic balance is not fully restored. Thus, LD accumulation represents a parallel adaptive response to HOG signaling to support cell growth under hyperosmotic conditions.

Mechanistically, LD accumulation during chronic stress is distinct from the acute phase. Acute osmotic shock primarily engages Lro1-dependent TAG production (Phan et al., 2025), whereas chronic stress relies on Dga1. This shift in enzymatic dependence suggests that chronic hyperosmotic stress activates a distinct lipid storage program, likely reflecting a transition from acute membrane slack buffering to long-term membrane adaptation.

Beyond the increase in LDs, persistent hyperosmotic stress did not cause widespread organelle remodeling, with the morphology of the ER and mitochondria being largely preserved, even in cells unable to form LDs. This specificity argues that LD accumulation does not occur as a secondary consequence of generalized stress. Instead, LD accumulation appears to support the full extent of hyperosmotic stress-triggered lipidome remodeling. Beyond the expansion of neutral lipids, we observed a shift in phospholipid composition, characterized by an elevation in PC and a concomitant reduction in PE. While the PE decrease occurs independently of LD formation, the magnitude of PC elevation is attenuated in cells lacking LDs. Our genetic and metabolic perturbations further demonstrate that increased PC contributes to fitness during chronic hyperosmotic stress, particularly when TAG synthesis is compromised. Together, these data support a model in which TAG synthesis and phospholipid remodeling both contribute to hyperosmotic stress adaptation, with LDs amplifying the increase in PC rather than driving the phospholipid changes as a whole. In the future, it will be interesting to investigate how lipid flux is rerouted during this response, including the source of the acyl chains channeled into TAG and the turnover of individual phospholipids, questions that will require metabolic labeling approaches. Nonetheless, our findings expand the role of LDs beyond neutral lipid storage, implicating them in membrane lipid remodeling during chronic environmental stress.

The selective requirement for Dga1 may be particularly important in this context. Unlike Lro1, which can generate TAG by transferring acyl chains from membrane phospholipids, including PC, Dga1 utilizes DAG and activated fatty acids (Oelkers et al., 2000). Preferential involvement of Dga1 during chronic stress may therefore promote TAG synthesis while preserving membrane phospholipid pools, facilitating lipid remodeling without depleting structural lipids. Consistent with this interpretation, TAG-deficient cells constitutively upregulate PC biosynthetic enzymes, yet remain compromised for growth under stress, suggesting that LD formation supports functional phospholipid adaptation beyond simple enzyme induction.

Interestingly, the enzymes driving these two responses appear to be controlled differently: Dga1 induction under stress involves a transcriptional component, whereas the increase in the PC biosynthetic enzymes Cho2 and Opi3, both during stress and upon TAG depletion, occurs without a corresponding change in mRNA and is therefore posttranscriptional. The molecular basis of this posttranscriptional regulation, and how it is engaged by both hyperosmotic stress and the loss of TAG synthesis, remains to be determined.

Consistent with previous observations that persistent hyperosmotic stress alters membrane physical properties such as fluidity, we observed reduced ER membrane protein mobility under these conditions (Laroche et al., 2001). Membrane fluidity changes are most commonly associated with enhanced lipid saturation and/or length, as well as ergosterol concentration (Renne and Ernst, 2023). Although we observed acyl-chain remodeling within individual phospholipid classes, the bulk saturation and length of membrane phospholipids were not substantially altered, pointing to a different underlying mechanism. The major physiological change upon hyperosmotic stress is elevated intracellular glycerol. Glycerol, and other sugars, can interact directly with phospholipid headgroups to alter membrane properties, decreasing lipid diffusion and increasing stiffness (van den Bogaart et al., 2007; Pocivavsek et al., 2011; Abou-Saleh et al., 2019; Schaich et al., 2020). This change could explain the observed decrease in membrane protein diffusion in the WT cells. We therefore speculate that glycerol acts on the membrane through two opposing effects: interactions at the phospholipid headgroups that reduce diffusion, and, when glycerol penetrates more deeply, intercalation into the bilayer that disrupts lipid packing and would instead tend to increase diffusion (Pereira and Hünenberger, 2008). PC, a fluidity-enhancing lipid with a bulky, weakly interactive headgroup (Seu et al., 2006; Dawaliby et al., 2011; Fajardo et al., 2011), engages glycerol less than PE and may buffer these interactions. In WT cells, the stress-induced rise in PC would favor controlled, headgroup-level interactions, yielding the net decrease in diffusion we observe. In TAG-deficient cells, where the PC increase is blunted, greater glycerol intercalation could counteract this stiffening, consistent with the failure of these cells to reduce membrane protein diffusion under stress. In this way, increased PC may help maintain membrane homeostasis during sustained hyperosmotic challenge.

More broadly, accumulation of osmolytes such as glycerol is a conserved feature of hyperosmotic adaptation across eukaryotes (Burg and Ferraris, 2008). Salt tolerance in plants is enhanced by elevated PC levels and is associated with TAG accumulation (Guo et al., 2019; Mueller et al., 2015; Corti et al., 2023). Renal epithelial cells, which experience chronic hyperosmotic stress, are enriched in both PC and TAG (Toback, 1984; Surma et al., 2021), and LD and PC accumulation are hallmarks of cancer cells subjected to mechanical and osmotic compression (Linke et al., 2024; Quan et al., 2025; Li et al., 2025). Our findings suggest that coordinated LD expansion and phospholipid remodeling may represent a conserved strategy for preserving membrane function during sustained osmotic imbalance.

## Materials and methods

### Yeast growth conditions

All strains used were derived from the BY4741 collection, with vectors sourced from Williams et al. (2024), and are listed in Table S1. Mutants were made either using homologous recombination as described in detail elsewhere, or by crossing and dissection using a Singer SporePlay + microscope, followed by genotype determination. Strains that had not previously been verified were verified by performing PCRs within the deleted genes.

Cells were grown at 30°C on YPD agar plates (10 g/l yeast extract, 20 g/l peptone, 2% glucose, 20 g/l agar) before being used for the described experiments. For all growth assays, cells were grown in SCD (6.7 g/l YNB, 0.6 g/l CSM) in 16-mm-diameter glass tubes at 30°C, 180–200 rpm, for a minimum of 14 h to allow them to enter the logarithmic phase. Cells were diluted to 0.05 OD_600 nm_ in 200 μl of the indicated media in a well of a flat-bottomed 96-well plate. The plate was incubated in a VANTAstar (BMG Labtech) at the indicated temperatures for the indicated times, with 30 s of vigorous shaking followed by OD_600 nm_ measurement every 15 min.

### Flow cytometry and analysis

Cells were grown to the logarithmic phase the day before they were due to be analyzed in SCD (16-mm-diameter glass tubes at 30°C, 180–200 rpm, for a minimum of 4 h), unless otherwise indicated. Overnight they were diluted into 25-mm-diameter glass tubes or conical flasks, such that they would grow to ∼0.05–0.5 OD_600 nm_ after 16 h at 30°C, 180–200 rpm. Samples were taken before treatment and stored on ice, then subjected to the indicated treatment with samples taken at the indicated times. These samples were then subject to flow cytometry analysis using a Fortessa (BD Biosciences) controlled by FACSDiva software (BD Biosciences) with a High-Throughput Sampler attachment set to record 10,000 events. Subsequent analysis was performed on FCS3.0 files using FlowJo (v10.10.0) to isolate bona fide single cells through gating, and the median relevant fluorescence value (either GFP or mCherry) for this population was recorded. From this median value, the median value of an unlabeled sample run alongside it was subtracted to obtain the final value.

### Microscopy and analysis

Cells were grown as for the flow cytometry experiments, with the exception that the overnight setup aimed for the cells to grow to between 0.2 and 0.5 OD_600 nm_. Samples were taken at the indicated treatment times and labeled with MDH (0.1 mM AutoDot, SM1000b; Abgent) or BODIPY 493/503 (1 µg/ml, Invitrogen) where indicated and taken straight for imaging without washing. Imaging was performed at room temperature on either a Zeiss Axio Observer with a 63× objective (Plan-APOCHROMAT 1.4) using a digital CMOS camera (ORCA Flash 4.0; Hamamatsu) and SlideBook 6.0 (3i) software, or Olympus FV3000 microscope with a 60× objective (Plan-APO N 1.4) using internal detectors and FV (Olympus) software.

Analysis and image preparation were performed using FIJI. For analysis, images of Ilv6-GFP, as well as MDH- and BODIPY-stained cells, underwent Z-projection using the standard deviation setting. Cho2-GFP, Opi3-GFP, and Sec63-GFP were imaged at the midsection. BODIPY-stained LDs were detected using ComDet (v.0.5.6) to isolate spherical particles and define their cross-sectional area in pixels, which was mathematically converted to diameter in Microsoft Excel. Detected LDs per cell were counted manually following ComDet particle identification, with budding cells counted as one cell if the bud was <50% of the size of the mother, and two separate cells if it was >50% the size of the mother. Any detected particle outside a cell was excluded from further analysis. Mitochondrial fragmentation was scored by eye, with cells having either completely elongated, tubular mitochondria; completely fragmented, punctate mitochondria; or a mixture of both.

### FRAP microscopy and analysis

Cells were prepared as for the above microscopy analysis, with the FRAP assays performed at 30^°^C on a SoRa spinning disk (Olympus) microscope with 60× objective (UPlanApo 1.5) and a Photometrics 95B camera using cellSens software. Cells were imaged for five frames before FRAP was performed in a stripe across the cell, and then, imaging proceeded for another 60 frames, each at 300-ms exposure. Sec63-GFP cells were imaged with a frame every 2 s, while HDEL-GFP had a frame every 0.5 s.

Analysis was performed as previously described (Shyu et al., 2019). Briefly, the intensities over time were taken from membranes subjected to FRAP, and not subjected to FRAP on the same cell using FIJI. The following equations were then used to define the values for each time point, normalizing for photobleaching, the initial fluorescence of the cell, and the lowest fluorescence of the bleached area.

$$\begin{array}{c} Weighted\;fluorescence=\\ \frac{\left(Fluorescence\;\left(FRAP\;region\right)-background\right)}{\left(Fluorescence\;\left(control\;region\right)-background\right)} \end{array}$$

$$\begin{array}{c} Normalized\;fluorescence=Weighted\;fluorescence\times \\ \frac{\left(Average\;preFRAP\;weighted\;fluorescence\;\left(FRAP\;region\right)-background\right)}{\left(Average\;preFRAP\;weighted\;fluorescence\;\left(control\;region\right)-background\right)} \end{array}$$

$$\begin{array}{c} Double\;normalized\;fluorescence=\\ \frac{\left(Normalized\;fluorescence\;\left(FRAP\;region\right)-Lowest\;fluorescence\right)}{\begin{array}{c} (Average\;preFRAP\;normalized\;fluorescence\;\left(FRAP\;region\right)\\ -Lowest\;fluorescence) \end{array}} \end{array}$$

From these data, the initial recovery rates (which show recovery independently of the mobile fraction) and the half-time recovery (which takes into account the mobile fraction) were calculated for each region subjected to FRAP using GraphPad Prism. The initial recovery rate was calculated by performing linear regression analysis to obtain the gradient during the initial stages of recovery where the average was linear (0–10 s for Sec63-GFP, 0–1.5 s for HDEL-GFP). The gradient was then converted into % recovery per second. The half-time recovery (T_1/2_) was calculated by fitting the entire recovery curve to an exponential plateau curve, yielding values for the Y intersect (Y0, set as 0), the maximum Y value (Ym), and a rate constant, K. The following calculation was then performed to obtain the X value in seconds for a Y value of 0.5 (i.e., halfway recovered). Values where the curve was a poor fit were excluded.

$$t\left(\frac{1}{2}\right)=\frac{\ln\left(\frac{\left(Ym-\frac{Ym}{2}\right)}{\left(Ym-Yo\right)}\;\right)}{-k}.$$

### Protein extraction and western blotting

Cells were grown as for the flow cytometry experiments, with the overnight growth occurring in 5–10 ml SCD in a conical flask. Cells were then diluted down to 0.2 OD_600 nm_ in SCD + 1M KCl, and samples of 2 OD_600 nm_ were harvested at the indicated times. UT samples were taken from the overnight culture prior to dilution. Samples were spun down in a benchtop centrifuge (8,000 rpm, 30 sec) and resuspended in 300 μl ice-cold 150 mM NaOH. After incubation for 10 min on ice, the cells were spun down again and resuspended in 100 μl lysis buffer (0.1M Tris-HCl, pH 6.8, 2% SDS). Samples were incubated at 65°C with shaking in an Eppendorf ThermoMixer F1.5 for 15 min, then brief spun to remove cell debris. The protein concentration of the supernatant was determined on a NanoDrop, and the samples were adjusted to the lowest value. 80 μl of sample was then mixed with 20 μl loading dye (50% glycerol, 0.05% bromophenol blue) and stored at −20°C prior to use.

For western blot analysis, ∼100 µg of protein samples was run on 4–20% Criterion TGX Precast SDS-PAGE gels (Bio-Rad) at 0.06 Amps per gel for 45 min. Blotting was performed using the Trans-Blot Turbo semidry system (Bio-Rad) onto PVDF membranes. Gel running and blotting were performed according to the manufacturer’s instructions. After blotting, membranes were blocked in 5% milk for 1 h, then incubated overnight with primary antibodies. Following primary antibody incubation, secondaries were applied for at least 1 h before imaging was performed using Western Lightning ECL Pro Enhanced Luminol Reagent (Revvity) on an Amersham Imager 600 (General Electric). Between each of these steps, membranes were twice rinsed with PBS-T (PBS with 0.1% Tween-20), followed by three 15-min washes. Primary antibodies were all diluted in PBS-T/1% milk, 0.0002% sodium azide and stored at 4°C between uses. Secondary antibodies were diluted 1:10,000 in PBS-T and used once. Primary antibodies used in this study were as follows: α-Dga1 (1:3,000, rabbit, homemade), α-Flag (1:2,000, mouse, A8592-2MG; Sigma-Aldrich), α-HA (1:2,000, rat, 3F10; Roche), α-Myc (1:1,000, mouse, 9E10; Thermo Fisher Scientific), α-tubulin (1:1,000, rat, sc-53030; Santa Cruz), α-PGK1 (1:5,000, mouse, 22C5D8; Thermo Fisher Scientific). Secondary antibodies used in this study were as follows: α-rabbit IgG (mouse, 211-032-171; Jackson ImmunoResearch), α-rat IgG (goat, 112-035-175; Jackson ImmunoResearch), α-mouse IgG (goat, 115-035-174; Jackson ImmunoResearch).

Western blot analysis was performed using FIJI, with the average band intensity determined by drawing a box around it and using the measure feature. Intensity was normalized to that of the loading control (tubulin or PGK1) to compensate for any errors in loading, then to the level of the untreated sample to account for differences between different membranes and imaging times.

### Lipidomic sample preparation

Cells were crashed out of frozen stocks onto an YPD agar plate and grown overnight at 30°C. Cells were then resuspended to ∼0.2 OD_600 nm_ in 2 ml SCD in a 16-mm-diameter tube and grown for 4 h at 30°C, 180 rpm. They were then diluted to 0.07 OD_600 nm_ in 5 ml SCD in a 25-mm-diameter tube. After a further 4-h growth at 30°C, 180 rpm, they were diluted into 250 ml SCD in a 1-liter conical flask so they would reach ∼0.4–0.8 OD_600 nm_ after 16 h (WT and hog1Δ: to 0.0008 OD_600 nm_, are1Δare2Δdga1Δlro1Δ: to 0.001 OD_600 nm_). In the morning, cells were inoculated in 250 ml SCD in 1-liter conical flasks at a concentration of 0.1 or 0.25 OD_600 nm_ for UT and KCl-treated samples, respectively, and returned to the incubator. After 3 h, equal OD_600 nm_ of all samples was taken and spun down using a JLA10.500 rotor (12 min, 4,000 rpm, 4°C). Pellets were resuspended in 10 ml ice-cold water, transferred to a 50-ml tube, and spun down again using a benchtop centrifuge (5 min, 3,900 rpm, 4°C). The pellet was resuspended in 1 ml of ice-cold water, and the OD_600 nm_ was determined. 20 OD of cells was transferred to individual precooled 1.5-ml tubes. For all except 1 tube, these were spun down again and the pellets flash-frozen in liquid nitrogen before long-term storage at −70°C. For the other sample, the volume in the tube was made up to 500 μl by the addition of ice-cold water and transferred to a 2-ml tube containing 0.5-ml 0.5-mm-diameter glass beads (Thistle Scientific). Tubes were sealed with parafilm and subjected to lysis by bead beating at 4°C (OMNI International Bead Ruptor Elite, 10 × 2.9m/s 1 min on, 3 min off). After lysis, 0.5 ml ice-cold water was added to the samples and mixed by pipetting, and 0.5 ml was transferred without beads to a new 2-ml tube, which was flash-frozen in liquid nitrogen. Samples were stored at −70°C or on dry ice until they were ready to undergo analysis.

### Lipidomic analysis

Lipidomic mass spectrometry analysis was performed by Lipotype GmbH. Lipids were extracted using chloroform and methanol (Klose et al., 2012). Samples were spiked with lipid class-specific internal standards prior to extraction. After drying and resuspending in MS acquisition mixture, lipid extracts were subjected to mass spectrometric analysis. Mass spectra were acquired on a hybrid quadrupole/Orbitrap mass spectrometer equipped with an automated nanoflow electrospray ion source in both positive and negative ion modes. Lipid identification was performed using LipotypeXplorer (Herzog et al., 2012) on unprocessed (*.raw format) mass spectra. For MS-only mode, lipid identification was based on the molecular masses of the intact molecules. The MSMS mode included the collision-induced fragmentation of lipid molecules, and lipid identification was based on both the intact masses and the masses of the fragments. Prior to normalization and further statistical analysis, lipid identifications were filtered according to mass accuracy, occupation threshold, noise, and background. Lists of identified lipids and their intensities were stored in a database optimized for the particular structure inherent to lipidomic datasets. Intensity of lipid class-specific internal standards was used for lipid quantification. Data are presented in either mol%, where the total molarity of the defined class adds up to 100%, or normalized pmol, where the picomoles of each lipid are normalized to account for the differing volumes of sample analyzed.

### Determination of mRNA levels by RT-qPCR

Cells were grown and treated as described above. 1 OD_600nm_ of cells was lysed per condition by bead beating using the settings described in the lipidomic analysis, and RNA was isolated using the Monarch Spin RNA isolation kit (NEB) according to the manufacturer’s instructions. Triplicate RT-qPCRs were performed using Luna Universal One-Step RT-qPCR Kit (NEB) according to the manufacturer’s instructions using QuantStudio 3 qPCR System (Applied Biosystems) using the Comparative Ct with Melt setting for primers targeted against the indicated mRNAs, and the housekeeping mRNA *TUB2* (Table S2). For each mRNA and condition, the difference in Ct was calculated between the mRNA of interest and *TUB2* (ΔCt). The difference between those values and the equivalent WT UT was calculated (ΔΔCt) and converted to a fold change in mRNA expression.

$$mRNA\;levels\;\left(fold\;change\right)=LOG_{2}\left(2^{\left(-\Delta \Delta \text{Ct}\right)}\right).$$

### Statistical analysis

All statistical analysis and graph generation were performed using GraphPad Prism, with error bars representing the standard error. For dual-variable grouped graphs (Fig. 2 B; Fig. 3, B, F, and G; Fig. 4, A, B, D, and E; Fig. S1, C–G, and I; Fig. S2, A, D, H, and L–N; Fig. S3, B, F–I, and Q; Fig. S4, B–H; Fig. S5, B, D–F, and H), two-way ANOVA between matched replicates was performed, followed by multiple comparisons analysis between samples of the same genotype and samples of the same treatment condition. For single-variable column graphs (Fig. 1, B, C, E, and F; Fig. 2, D and E; Fig. 3, D and E; Fig. S1 B; Fig. S2, C, G, J, and K; and Fig. S3, D, E, I, and J), one-way ANOVA with matched replicates was performed, followed by multiple comparisons between all samples. Paired *t* tests were performed for Fig. S1 J, Fig. S3, M–P; and Fig. 7, C–F, I, and J. For Fig. S3 L, a one-sample *t* test against an expected mean of 1 was performed. For Fig. 6 E and Fig. S5 K, Tukey’s multiple comparisons tests were performed.

### Online supplemental material

Fig. S1 shows the utility of using Pln1-mCherry as a proxy for LD formation, and how levels of this protein respond to a variety of cellular stresses. Fig. S2 explores the role of the seipin complex, as well as TAG and STE synthesis in regulating LD accumulation upon chronic hyperosmotic stress. It further looks at the roles of endocytic and lipid trafficking proteins reported to be involved in a LD response to acute hyperosmotic stress. Fig. S3 examines the roles of the DAG and acyl-FA machinery in LD accumulation and the regulation at the protein and mRNA level of the proteins which synthesize TAGs and STEs. Fig. S4 shows the effect on Pln1-mCherry levels following hyperosmotic stress of manipulating the HOG and other stress-responsive signaling pathways. Fig. S5 contains more details from the lipidomic experiment which are presented in Fig. 5, alongside an examination of Cho2 and Opi3 mRNA regulation. Tables S1 and S2 show the strains and primers used in this study, respectively.

## Supplementary Material

Review History

Table S1 shows strains used in this study.

Table S2 shows primers used in this study.

SourceData FS3 is the source file for Fig. S3.

SourceData FS5 is the source file for Fig. S5.

## Data Availability

All data used in this study are available upon request.
