# Supplementary material for: Lipid remodeling enables adaptation to chronic hyperosmotic stress
Source: J Cell Biol. 2026 Oct 8;225(11):e202603154. doi: 10.1083/jcb.202603154 (PMC13648197; doi:10.1083/jcb.202603154)
Supplement: Supplementary file 1 [file jcb_202603154_review_history.pdf]

JCB

Lipid remodelling enables adaptation to chronic hyperosmotic stress

Thomas Williams, Christian Klose, Robert Ernst, and Pedro Carvalho

Corresponding Author(s): Pedro Carvalho, University of Oxford and Thomas Williams, University of Oxford

Review Timeline:

Submission Date:	2026-03-24
Editorial Decision:	2026-04-24
Revision Received:	2026-07-22
Editorial Decision:	2026-08-07
Revision Received:	2026-09-02

Monitoring Editor: Sarah Cohen

Scientific Editor: Andrea Marat

Transaction Report:

(Note: With the exception of the correction of typographical or spelling errors that could be a source of ambiguity, letters and reports are not edited. The original formatting of letters and referee reports may not be reflected in this compilation.)

DOI: https://doi.org/10.1083/jcb.202603154

April 24, 2026

Re: JCB manuscript #202603154

Pedro Carvalho
University of Oxford

Dear Dr. Carvalho,

Thank you for submitting your manuscript entitled "Lipid remodeling enables adaptation to chronic hyper osmotic stress" to the Journal of Cell Biology. The manuscript was assessed by expert reviewers, whose comments are appended to this letter. You will see that while the reviewers found the question and observations interesting, all had significant concerns about the level of mechanistic insight provided. In addition, Reviewers 1 and 2 also requested further development of the physiological significance of increased PC levels. For example, Reviewer 1 asks about the source of acyl chains shunted into lipid droplets and the role of elevated PC levels in the stress response. Reviewer 2 states that the causal link between TAG accumulation and PL remodeling is not sufficiently established, and that the explanation provided for the physiological benefit of PC increase is largely speculative. Altogether, while your manuscript is intriguing, the points raised by the reviewers seem more substantial than can be addressed in a typical revision period. If you wish to expedite publication of the current data, it may be best to pursue publication at another journal.

Given interest in the topic, we would be open to resubmission to JCB of a significantly revised and extended manuscript that fully addresses the reviewers' concerns and is subject to further peer-review. If you feel that you can address the reviewers' comments, we strongly encourage you to submit a revision plan for approval before working on revision experiments. This plan should expand on the mechanism underlying lipid remodeling, for example by identifying the source of acyl chains shunted into TAG or further examining the regulation of key lipid synthesis enzymes. You should also plan to test the physiological function of the PC increase, for example in maintaining membrane homeostasis or mitochondrial fitness. The additional lipidomics experiments suggested by Reviewer 3 would not be a high priority if these are demanding. Please note that priority and novelty would be reassessed at resubmission.

Regardless of how you choose to proceed, we hope that the comments below will prove constructive as your work progresses. We would be happy to discuss the reviewer comments further once you've had a chance to consider the points raised in this letter. You can contact the journal office with any questions at cellbio@rockefeller.edu. If you prefer to transfer your manuscript to another journal such as Life Science Alliance (LSA), our academic editor-led, open access journal launched as a collaboration between RUP, EMBO Press and Cold Spring Harbor Press, or JCS or MBoC, we would be pleased to facilitate the transfer.

Thank you for thinking of JCB as an appropriate place to publish your work.

Sincerely,

Sarah Cohen, PhD
Monitoring Editor

Andrea L. Marat, PhD
Deputy Editor

Journal of Cell Biology

Reviewer #1 (Comments to the Authors (Required)):

Williams et al describe the effects of long-term hyperosmotic stress in the lipid metabolic program of budding yeast cells. They document two major changes, driven by potassium chloride addition: (a) lipid droplets (LDs) and triglyceride accumulate following sustained growth in KCL, in a manner that depends primarily on the DGAT Dga1, and (b) phosphatidylcholine (PC) levels increase, driven by increases of the levels of the PC-synthesizing enzymes, while the levels of phosphatidylethanolamine (PE) decrease. The LD accumulation is not controlled by the HOG pathway, but loss of HOG leads to a further increase of LDs. Finally, the authors also show PC levels in LD-deficient cells decrease. The authors conclude that lipid metabolic changes are part of the response to hyperosmotic stress.

LDs often accumulate in response to perturbations of normal growth and disease states but the mechanisms remain not well

understood. The topic of this manuscript is therefore of interest. The authors present largely well-executed experiments and report some novel and interesting observations - for example, the effect of the HOG pathway inactivation on the LDs in cells under salt stress and the changes in phospholipid classes. However, the current data appear too preliminary to support a coherent hypothesis or a model. The physiological significance of LD build-up and the phospholipid alterations during the chronic adaptation to hyperosmotic stress is not clear to me. Based on plate assays, the authors argue that triglyceride synthesis is required under these conditions for "cellular fitness", but the mechanism is not addressed. What is the source of the acyl chains shunted into droplets? What is the role of the elevated PC levels? At the very least, the authors should test their hypotheses that PC maintains membrane homeostasis or mitochondrial function during stress.

The relationship between the long-term (described here) and acute (Phan et al 2025) responses to hyperosmotic stress could be defined better. The authors argue that they are distinct, based on the different requirement of acyl-transferases. However, the assays are different, Phan et al followed only newly formed LDs which were small and cortical, while the authors follow total LDs in 3h. Cell surface recovers 40% after the acute response to stress - does cell surface recovers further at later points and could Dga1 contribute? Also, the osmotic insults are different (sorbitol - inert and non-ionic while KCl introduces also ionic stress) and it is not clear whether the acyl-transferases have different roles under these conditions.

Other points:

- The plate growth spot assays are not wholly convincing, and in some cases the observed differences appear to be, at best, marginal: in Fig. 2A, the dga1 lro1 mutant does show a growth delay (although I would not call this a "severe defect") but in other figures (5C and 5D) the dga1 lro1 does not show an apparent growth defect. The growth curves in Fig. S5A and B document a modest defect when comparing KCl between wt and dga1 lro1. Also, I cannot really see the effect of the Pct1 deletion on the choline supplemented cells (Fig. 5D), which is critical to the claim of the beneficial role of PC during hyperosmotic adaptation.
- Nem1 and Faa1/4 depletion - not sure if this experiment adds much.
- "Thus, chronic hyperosmotic stress remodels the most abundant phospholipids and alters overall phospholipid composition." The first part of this statement is not correct: there is no evidence of remodelling of PC and/or PE species here.
- Lines 265-266, should read Figure 5C, not 5D.

Reviewer #2 (Comments to the Authors (Required)):

Summary: This paper investigate how yeast adapt to chronic hyperosmotic stress through significant lipid remodeling. The authors demonstrate that long-term exposure to high osmotic pressure triggers the accumulation of LDs, primarily driven by Dga1-mediated TAG synthesis. The response is distinct from the HOG signaling pathway that promotes glycerol production in response to hyperosmotic stress. Inhibition or inactivation of the Hog pathway increased LDs even more than the WT in response to KCL treatment. Therefore, they suggest that activation of the HOG pathway limits the increase in LDs in response to hyperosmotic stress. Lipidomic analysis revealed a shift in membrane composition from PE to PC, which helps maintain membrane fluidity. The study offers a genetic and lipidomic analysis of how yeast cells adapt to chronic osmotic stress and concludes that LD accumulation and phospholipid remodeling are essential for cellular fitness during prolonged osmotic stress adaptation.
Overall, the works provide significant impact by demonstrating that chronic hyperosmotic stress triggers sustained lipid remodeling. A major strength is the integrative methodology combining high-throughput screening with lipidomic analysis to characterize the adaptive shift from PE to PC. The first half of the paper presents a rigorous characterization of the cellular and genetic response to KCL and the changes in LDs is strong. Robust genetic dissection strengthens the claims establishing Dga1-driven TAG synthesis as essential for maintaining cellular fitness. The second half, which focuses on lipid remodeling in response to KCL is significantly less cohesive. The causal link between TAG accumulation and PL remodeling is not sufficiently established. A notable weakness is that the study lacks a defined molecular mechanism explaining how key enzymes (like Dga1 and Opi3) are actually upregulated during stress. Furthermore, that explanation for the physiological benefits of PC increase is largely speculative and not relying on direct measurement or experimental framework.
The major limitation is the lack of mechanism for enzymatic regulation and the marginal growth rescue observed in choline supplementation. While the study presents a robust genetic framework for the "chronic" adaptation to osmotic stress, the conclusions remain speculative and lack direct measurements to confirm their model. Explaining why these changes are triggered and how they rescue the cell needs to be investigated.
Below are some specific points for consideration.
- The authors conclude that LD-driven TAG synthesis and PL remodeling are functionally coupled. However, Figure 4F shows that LD-KO cells show that the increase in PC and decrease in PE still occurs in absence of LDs.
- Choline supplementation only slightly improved the growth of Dga1Lro1 KO. How does that fit the major claim that PC levels modulate the fitness of TAG deficient cells? Lipid levels after supplementation are not tested directly.
- Regulation of PC enzymes is not clear. The levels of these enzymes are already high in the mutant and don't increase further in response to stress. This suggests basal lipid sensing defects rather than specific response to osmotic stress. The mechanism of upregulation was not tested.
- The core explanation why PC levels increase to counteract glycerol-induced membrane remodeling is theoretical and no

experimental evidence presented to support this.
- It is not clear if LD accumulation is a specific programmed adaptive response or a symptom of cell failure.
Other points:
- There are some inconsistencies in the plating assays in particular in the growth sensitivity of dga1lro1 KO for example in 5C compared to 2A that are not addressed.
- Line 266: 5C not 5D.
- Experiments should be explained in more detail in the text. For instance, the part where mutants in the HOG pathway are used is not sufficiently explained.
- In general, the trends in changes in LDs should be explained more accurately. In some places there are trends or changes that are not significant but worth commenting on. Also accurately describing the results as to what is changing and what is trending up or down or slight variations vs no change should be more carefully and accurately described.
- The paper lacks a does response curve for KCL. 1M concentration shows sever growth defect in Fig 2a but not in Fig 5 and then used in growth curves in Fig s5a. It is not clear if these pathways are essential for survival under moderate concentrations of KCL.
- The authors distinguish their work from previous studies on "acute" osmotic shock by focusing on "chronic" however the definition of chronic is not clear. They show that LD signal peaks at 3hr and then goes down steadily over the next few hours. In yeast 3hr represent s two generational cycles, calling this response chronic needs to be more carefully considered.

Reviewer #3 (Comments to the Authors (Required)):

The manuscript by Williams et al. describes a role for lipid droplet biogenesis in adaptation to hyperosmotic stress. Using a recently developed semi high throughput screen, the authors quantify lipid droplet abundance under several stress conditions and identify hyperosmotic stress as a trigger for droplet accumulation in S. cerevisiae. A combination of yeast genetics and growth assays leads them to conclude that triacylglyceride containing droplets are formed mainly through the activity of the diacylglycerol acyl transferase Dga1 (DGAT). The authors also explore the involvement of the classic hyperosmotic stress response pathway and find only indirect connections. Finally, lipidomics measurements reveal lipid remodeling under hyperosmotic stress, and the authors test the contribution of the phosphatidylethanolamine (PE) and phosphatidylcholine (PC) biosynthetic pathways. Overall, the manuscript is well written, the data are well controlled, and the statistical analyses meet the standards of Journal of Cell Biology.
The study is well executed and the finding that Dga1 drives lipid droplet biogenesis under high osmolarity conditions is compelling. However, the conclusions drawn from the lipid remodeling data are currently speculative and not fully supported by the presented evidence. Several major points need to be addressed to strengthen the manuscript.
Figure 2 currently contains three redundant methods for quantifying Dga1 levels, while potentially informative data such as the dependence on FAA1/4 or Nem1 are relegated to the supplement. It would improve clarity to consolidate the Dga1 quantification into a single, most informative assay and to move the FAA1/4 and Nem1 data to the main figure.
Figure 4: The data on hog1 cells are not very consistent. The large increase in droplet number and size does not match the measured TAG levels. Is the increased number and size of droplets directly dependent on the 3 mol % increase in sterol esters. The description that "LDs in hog1 cells are compositionally distinct" are not satisfactory.
The same figure presents only mol % changes in lipid species, which limits the impact of the analysis. The manuscript would benefit from expanding the lipidomics data to include fatty acid chain length and saturation profiles, and from discussing how these parameters might affect membrane properties and droplet formation. The current description of the involvement of the PE/PC biosynthetic pathways remains largely descriptive.
Similarly, the growth assay results shown in Figure 5 are difficult to interpret without knowledge of how the lipid composition changes in the cho2, opi3, or other double mutant backgrounds. Performing lipidomics on each mutant under the same stress conditions-or at least providing a brief summary of their baseline lipid profiles-would allow readers to link growth phenotypes to specific lipid alterations.
Figure 6 merely quantifies Cho2 and Opi3 protein levels. This information could be incorporated into Figure 5 as an additional panel, or, if retained as a separate figure, should be complemented with functional data (e.g., enzyme activity assays) to justify a stand alone presentation.
To substantiate the claim that lipid remodeling actively drives droplet biogenesis, flux measurements (e.g., pulse chase labeling with stable isotopes) would be highly informative. While such experiments may be beyond the scope of a rapid revision, acknowledging this limitation in the discussion would temper overly speculative statements.
In summary, the data convincingly demonstrate Dga1 dependent lipid droplet formation under hyperosmotic stress, but the current lipid remodeling evidence does not fully support the broader mechanistic conclusions. Minor figure restructuring, additional lipid composition data for key mutants, and a more cautious interpretation of the lipidomics results will substantially improve the manuscript.

Reviewer #1

"What is the source of the acyl chains shunted into droplets? What is the role of the elevated PC levels? At the very least, the authors should test their hypotheses that PC maintains membrane homeostasis or mitochondrial function during stress."

We thank the reviewer for these questions, which we have addressed with new experiments. To test the role of elevated PC, we examined the two possibilities we had previously raised only as speculation. First, we now show that mitochondrial morphology and dynamics (fragmentation and subsequent recovery) in TAG-deficient cells were indistinguishable from WT under hyperosmotic stress (Figure 4), arguing against a mitochondrial basis for the PC requirement. Second, and in contrast, we found a direct effect on ER membrane properties: diffusion of the ER membrane protein Sec63-GFP decreased upon stress in WT cells but not in cells unable to synthesise TAG (Figure 7). We interpret this as indicative that LD formation, through its support of PC synthesis, contributes to the adaptation of membrane properties during sustained stress. The text has been revised accordingly, and discussed in the 4th paragraph of the discussion.

Regarding the source of the acyl chains, we performed acyl-chain-resolved analysis of the TAGs (Figure 5, Figure S5). Because the phospholipid acyl-chain profiles of WT and LD-deficient cells are similar after stress, acyl-chain remodelling does not appear to be the primary functional consequence of TAG accumulation; we therefore did not pursue the source of the acyl chains further, and we now note this explicitly as a limitation in the 3rd paragraph of the discussion (see also our response to Reviewer 3 regarding flux measurements).

"The relationship between the long-term (described here) and acute (Phan et al 2025) responses to hyperosmotic stress could be defined better. […] the assays are different […] Cell surface recovers 40% after the acute response to stress - does cell surface recovers further at later points and could Dga1 contribute? Also, the osmotic insults are different (sorbitol - inert and non-ionic while KCl introduces also ionic stress) […]"

We thank the reviewer for raising this point. We have clarified this distinction in several ways. We have added a paragraph at the start of the 3rd section of the results defining the acute and chronic phases, and we now present additional genetic evidence that the LDs formed during chronic stress differ from those of the acute response: beyond their dependence on Dga1 rather than Lro1, they show no dependence on endocytosis or on Osh2/3-mediated lipid transfer (Figure S2). On the question of cell surface recovery, published work has not examined our timescales directly, but cell volume remains stable up to 45 minutes post-stress (Saulou-Bérion et al.), and the progressive decline in Hog1 activity — which is triggered by reduced membrane tension — indicates that tension is restored over time rather than continuing to fall; we therefore do not expect further surface recovery at the later timepoints we study. On the nature of the osmolyte, the non-ionic osmolyte sorbitol also induces Pln1-mCherry

accumulation in WT cells (Figure S1J), and the pattern of response is similar between sorbitol and KCl treatment with neutral lipid enzyme mutants (Figure S2L) indicating the response is not specific to ionic stress.

"The plate growth spot assays are not wholly convincing, and in some cases the observed differences appear to be, at best, marginal […] I cannot really see the effect of the Pct1 deletion on the choline supplemented cells (Fig. 5D), which is critical to the claim […]"

We thank reviewer for this point and agree that the spot assays were difficult to interpret quantitatively. We have therefore re-assessed cell proliferation throughout the manuscript using liquid growth curves. These are extremely reproducible and allow direct quantitative comparisons. These confirm that choline supplementation substantially rescues the growth of the TAG-deficient mutant under stress, and that this rescue depends on Pct1 (Figure 6B-D). We have also modified the text so that it more accurately describes the dga1Δlro1Δ growth phenotypes.

"Nem1 and Faa1/4 depletion - not sure if this experiment adds much."

We note that Reviewer 3 found these experiments informative and recommended promoting them to a main figure, whereas Reviewer 1 questioned their value. As a balance between these views we have retained them in the supplement and clarified their purpose in the text: by restricting the availability of Dga1's substrates (diacylglycerol and activated fatty acids), they reinforce the central conclusion that Dga1 drives LD accumulation during chronic stress.

"'Thus, chronic hyperosmotic stress remodels the most abundant phospholipids and alters overall phospholipid composition.' The first part of this statement is not correct: there is no evidence of remodelling of PC and/or PE species here."

We agree with the reviewer. We now present acyl-chain-resolved analysis of PE and PC (Figure 5, Figure S5). These data indicate that the PE pool shifts toward longer species through preferential depletion of shorter ones while the PC increase is driven in part by the emergence of a 16:0_16:1 species absent from untreated cells. We have revised the text to more accurately reflect the data.

"Lines 265-266, should read Figure 5C, not 5D."

This data has now been replaced and reorganised. We thank the reviewer for spotting the error.

Reviewer #2

"The authors conclude that LD-driven TAG synthesis and PL remodeling are functionally coupled. However, Figure 4F shows that LD-KO cells show that the increase in PC and decrease in PE still occurs in absence of LDs."

We thank the reviewer and agree that our original wording overstated this relationship. Our data in fact distinguish two arms of the phospholipid response: the stress-induced fall in PE occurs to a comparable extent in LD-deficient cells and is therefore LD-independent, whereas the rise in PC is attenuated — though not abolished — when LDs are absent (Figure 5D). We have revised the model throughout: rather than describing TAG synthesis as driving phospholipid remodelling, we now describe LDs as amplifying the PC arm of an otherwise LD-independent response. We have removed the term "functionally coupled" and rewritten the relevant statements in the Abstract, Results, and Discussion.

"Choline supplementation only slightly improved the growth of Dga1Lro1 KO. How does that fit the major claim that PC levels modulate the fitness of TAG deficient cells? Lipid levels after supplementation are not tested directly."

Using the more quantitative liquid growth curves, the rescue by choline is substantial rather than minor and it depends on Pct1 (Figure 6B-D) establishing that the effect is mediated by PC synthesis. We have not measured lipid levels directly after supplementation; however, the Pct1-dependence of the rescue demonstrates that the benefit depends on Kennedy-pathway PC synthesis and we now state this interpretation and its basis explicitly in the text.

"Regulation of PC enzymes is not clear. The levels of these enzymes are already high in the mutant and don't increase further in response to stress. This suggests basal lipid sensing defects rather than specific response to osmotic stress."

This is an important point. We agree that the constitutive elevation of Cho2 and Opi3 in the TAG-deficient mutant reflects an altered basal state rather than a stress-specific induction and we now describe it in those terms: loss of TAG synthesis appears to engage PC biosynthesis constitutively, consistent with cells compensating for an impaired capacity to remodel lipids. We have examined the underlying regulation and find that the increase is not transcriptional (Figure S5K). We performed further experiments measuring protein stability changes of Cho2-GFP using cycloheximide chases, but found these results to be inconclusive so have not included them in the manuscript. We are happy to share them (below) with the reviewers and incorporate if reviewers and/or editor find it appropriate:

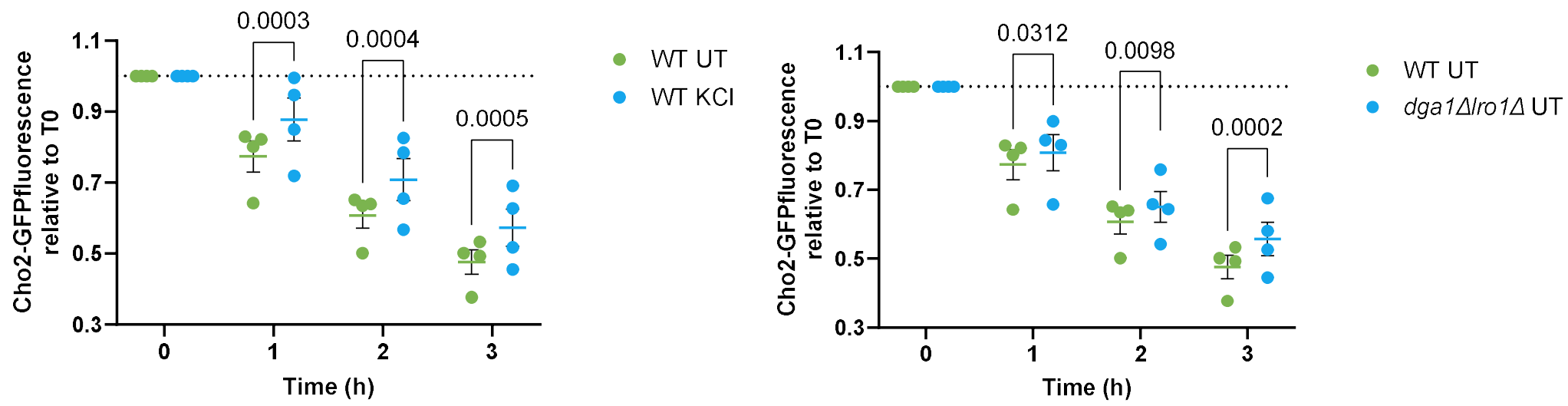

"It is not clear if LD accumulation is a specific programmed adaptive response or a symptom of cell failure."

We thank the reviewer for raising this important point. We have clarified the manuscript to distinguish LD accumulation as a specific adaptive response from nonspecific cellular failure. Several observations argue against LD accumulation being a symptom of cellular collapse. First, the response is reversible: upon removal of hyperosmotic stress, Pln1-mCherry levels return close to baseline within 3 h, indicating that LD accumulation is dynamically regulated by the osmotic environment rather than reflecting irreversible collapse. Second, although mitochondria fragment during the acute phase of hyperosmotic stress (as observed for other stresses like heat shock), this resolves within 1 h and at the chronic 3 h time point both ER and mitochondrial morphology are largely preserved in WT and TAG-deficient cells. Thus, sustained LD accumulation is not associated with persistent, widespread organelle dysfunction. Moreover, we now make clear that the similar organelle morphology of WT and TAG-deficient cells does not imply that TAG synthesis is dispensable. Instead, it shows that the reduced fitness of TAG-deficient cells is not caused by gross ER or mitochondrial collapse. The adaptive requirement for TAG synthesis is supported by the impaired growth of TAG-deficient cells under chronic hyperosmotic stress, their attenuated PC remodelling and their defective change in ER membrane protein mobility. We have revised the text around Figure 4 accordingly.

"The paper lacks a dose response curve for KCL. […] It is not clear if these pathways are essential for survival under moderate concentrations of KCL."

We include dose response data for Pln1-mCherry levels in Figure S1H, showing that the response is time and concentration dependent. We have further performed growth assays across a variety of KCl concentrations for the *dga1Δlro1Δ* mutant. It becomes progressively more sensitive as the stress increases. Given the volume of data already in the manuscript, we have included this in the response rather than adding it to the figures, but we are happy to incorporate it if reviewers and/or editor find it appropriate.

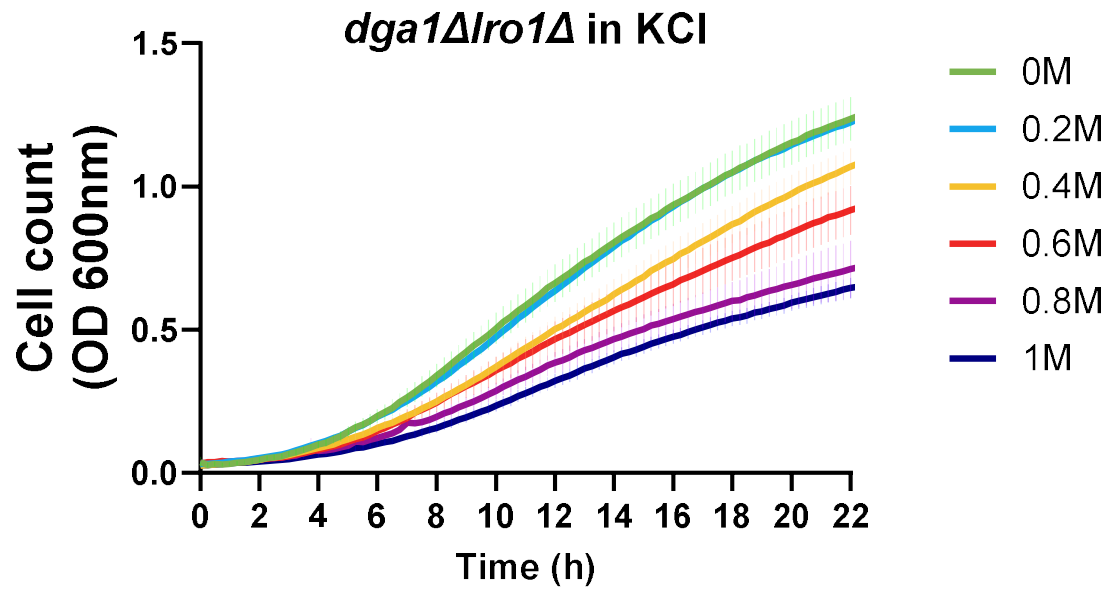

"the definition of chronic is not clear. They show that LD signal peaks at 3hr and then goes down steadily [...] In yeast 3hr represents two generational cycles, calling this response chronic needs to be more carefully considered."

We agree with the reviewer and have clarified our use of "chronic." The response is not transient: LD accumulation persists for at least 24 hours (Figure 1A&B), well beyond the 3–4 hour peak, and we define the chronic phase by cells re-establishing a proliferative steady state under sustained stress rather than by the peak itself. We note that the signal remains elevated throughout this period rather than declining to baseline. Importantly, the LD accumulation upon hyperosmotic conditions is completely and quickly reversed upon removal of the stress (see Figure 1C). We have revised the manuscript to make the basis for the distinction explicit, adding a new explanatory paragraph at the start of the third results section.

"the part where mutants in the HOG pathway are used is not sufficiently explained" / "trends in changes in LDs should be explained more accurately"

We have expanded the description of the HOG-pathway mutants in the figure legends, and additional mutants we have since used in the same figure, explaining the rationale for each. We have also revised the Results throughout to describe the LD data more precisely, distinguishing statistically significant changes from non-significant trends and noting the latter where biologically relevant, e.g. “the increase in PC was attenuated in *LD*Δ cells relative to WT and trended slightly lower in *hog1Δ* (Figure 5D)”

Reviewer #3

"Figure 2 currently contains three redundant methods for quantifying Dga1 levels [...] It would improve clarity to consolidate the Dga1 quantification into a single, most informative assay and to move the FAA1/4 and Nem1 data to the main figure."

We have consolidated the Dga1 quantification, retaining the microscopy data in the main figure and moving the Western blot and flow cytometry analyses to the supplement. As noted in our response to Reviewer 1, the Faa1/4 and Nem1 data were considered to add little value by Reviewer 1; we have retained them in the supplement as a balance between the two assessments, with their rationale now clarified in the text.

"The data on hog1 cells are not very consistent. The large increase in droplet number and size does not match the measured TAG levels. […] The description that 'LDs in hog1 cells are compositionally distinct' are not satisfactory."

We have addressed this with additional quantitative analysis. Using absolute (pmol-normalised) measurements, we now show the changes in the abundance of detected lipids (Figure S5A). We further characterise the neutral lipid composition more precisely (Figure S5B): stressed WT cells are more TAG-rich than the other conditions, and the *hog1Δ* cells differ in their TAG/STE balance. The apparent mismatch between LD number/size and total TAG mol% is consistent with these compositional differences, which can affect lipid packing and therefore Pln1 binding and BODIPY staining. We have rewritten the "compositionally distinct" statement to describe the specific differences rather than asserting the conclusion.

"The same figure presents only mol % changes in lipid species, which limits the impact of the analysis."

We have added absolute lipid quantification (pmol normalised to OD) for the major lipid classes and for the PE and PC species (Figure 5, Figure S5).

"The manuscript would benefit from expanding the lipidomics data to include fatty acid chain length and saturation profiles […]"

We thank the reviewer for this comment. We have now added acyl-chain length and saturation profiles for total phospholipids, TAG, PE, and PC (Figure 5, Figure S5; see our response to Reviewer 1). We further note that the phospholipid acyl-chain profiles of WT and LD-deficient cells are similar after stress suggesting that acyl-chain remodelling is unlikely to underlie the proliferation defect we observe.

"the growth assay results shown in Figure 5 are difficult to interpret without knowledge of how the lipid composition changes in the cho2, opi3, or other double mutant backgrounds."

We agree with the reviewer that these growth phenotypes are difficult to link to specific lipid changes without composition data for each background. As the choline-rescue experiment

and its Pct1-dependence (Figure 6B-D) now establish the contribution of PC more directly, and since we do not have the lipidomics analysis required to interpret the results we decided to remove the panels assessing phenotypes of PE and PC synthesis knock-out mutants.

"Figure 6 merely quantifies Cho2 and Opi3 protein levels. This information could be incorporated into Figure 5 […]"

We have re-ordered the figures as suggested.

"flux measurements (e.g., pulse chase labeling with stable isotopes) would be highly informative. While such experiments may be beyond the scope of a rapid revision, acknowledging this limitation in the discussion would temper overly speculative statements."

We agree. We now note in the Discussion that metabolic labelling approaches, such as pulse-chase, would be informative for establishing how lipid flux is rerouted during adaptation, including the source of the acyl chains channelled into TAG and the turnover of individual phospholipids. As our expanded analysis indicates that acyl-chain composition is not the critical output of TAG accumulation, we regard these as a valuable direction for future work rather than a requirement for the present study, and have acknowledged this explicitly in the text.

August 7, 2026

RE: JCB Manuscript #202603154R-A

Pedro Carvalho
University of Oxford

Dear Dr. Carvalho:

Thank you for submitting your revised manuscript entitled "Lipid remodelling enables adaptation to chronic hyperosmotic stress". Your study has been addressed by one of the original reviewers who is supportive of publication pending addressing their final concern. The other two reviewers were unfortunately not available, however we agree that you have thoroughly addressed all the reviewer points. Therefore we would be happy to publish your paper in JCB pending final revisions to meet the reviewer's question and necessary for our formatting guidelines (see details below).

To avoid unnecessary delays in the acceptance and publication of your paper, please read the following information carefully.

A. MANUSCRIPT ORGANIZATION AND FORMATTING:

Full guidelines are available on our Instructions for Authors page, https://jcb.rupress.org/submission-guidelines#revised.
Submission of a paper that does not conform to JCB guidelines will delay the acceptance of your manuscript.

1) Text limits: Character count for Articles is < 40,000, not including spaces. Count includes abstract, introduction, results, discussion, and acknowledgments. Count does not include title page, figure legends, materials and methods, references, tables, or supplemental legends.

2) Figures limits: Articles may have up to 10 main text figures.

3) Figure formatting: Scale bars must be present on all microscopy images, including inset magnifications. Molecular weight or nucleic acid size markers must be included on all gel electrophoresis. Aspect ratios of images may not be altered.

4) Statistical analysis: Error bars on graphic representations of numerical data must be clearly described in the figure legend. The number of independent data points (n) represented in a graph must be indicated in the legend. Statistical methods should be explained in full in the materials and methods. For figures presenting pooled data the statistical measure should be defined in the figure legends. Please also be sure to indicate the statistical tests used in each of your experiments (either in the figure legend itself or in a separate methods section) as well as the parameters of the test (for example, if you ran a t-test, please indicate if it was one- or two-sided, etc.). Also, if you used parametric tests, please indicate if the data distribution was tested for normality (and if so, how). If not, you must state something to the effect that "Data distribution was assumed to be normal but this was not formally tested."

5) Abstract and title: The abstract should be no longer than 160 words and should communicate the significance of the paper for a general audience. The title should be less than 100 characters including spaces. Make the title concise but accessible to a general readership.

6) Materials and methods: Should be comprehensive and not simply reference a previous publication for details on how an experiment was performed. Please provide full descriptions in the text for readers who may not have access to referenced manuscripts.

7) All antibodies, cell lines, animals, and tools used in the manuscript should be described in full, including accession numbers for materials available in a public repository such as the Resource Identification Portal. Please be sure to provide the sequences for all of your primers/oligos and RNAi constructs in the materials and methods. You must also indicate in the methods the source, species, and catalog numbers (where appropriate) for all of your antibodies. Please also indicate the acquisition and quantification methods for immunoblotting/western blots.

8) Microscope image acquisition: The following information must be provided about the acquisition and processing of images:
a. Make and model of microscope
b. Type, magnification, and numerical aperture of the objective lenses
c. Temperature
d. Imaging medium
e. Fluorochromes
f. Camera make and model
g. Acquisition software

h. Any software used for image processing subsequent to data acquisition. Please include details and types of operations involved (e.g., type of deconvolution, 3D reconstitutions, surface or volume rendering, gamma adjustments, etc.).

9) References: There is no limit to the number of references cited in a manuscript. References should be cited parenthetically in the text by author and year of publication. Abbreviate the names of journals according to PubMed.

10) Supplemental materials: There are strict limits on the allowable amount of supplemental data. Articles may have up to 5 supplemental figures. Please also note that tables, like figures, should be provided as individual, editable files. A summary of all supplemental material should appear at the end of the Materials and methods section.

11) eTOC summary: A ~40-50-word summary that describes the context and significance of the findings for a general readership should be included on the title page. The statement should be written in the present tense and refer to the work in the third person.

12) Conflict of interest statement: JCB requires inclusion of a statement in the acknowledgements regarding competing financial interests. If no competing financial interests exist, please include the following statement: "The authors declare no competing financial interests." If competing interests are declared, please follow your statement of these competing interests with the following statement: "The authors declare no further competing financial interests."

13) ORCID IDs: ORCID IDs are unique identifiers allowing researchers to create a record of their various scholarly contributions in a single place. Please note that ORCID IDs are now *required* for all authors. At resubmission of your final files, please be sure to provide your ORCID ID and those of all co-authors.

14) A separate author contribution section following the Acknowledgments. All authors should be mentioned and designated by their full names. We encourage use of the CRediT nomenclature.

Please note that JCB now requires authors to submit Source Data used to generate figures containing gels and Western blots with all revised manuscripts. This Source Data consists of fully uncropped and unprocessed images for each gel/blot displayed in the main and supplemental figures. For assays performed using capillary electrophoresis and/or immunoassay-based detection, authors should instead provide the electropherogram graph(s) for each experiment, plotting fluorescence/chemiluminescence intensity vs. molecular weight/size. Please be sure to provide one Source Data file for each figure gels, blots, and/or capillary electrophoresis assays along with your revised manuscript files. File names for Source Data figures should be alphanumeric without any spaces or special characters (i.e., SourceDataF#, where F# refers to the associated main figure number or SourceDataFS# for those associated with Supplementary figures). For traditional gels and blots, the lanes of the gels/blots should be labeled as they are in the associated figure, the place where cropping was applied should be marked (with a box), and molecular weight/size standards should be labeled wherever possible. For capillary electrophoresis assays, each trace in the graph should be color-coded and labeled to indicate which protein, gene, or sample is being measured (please try to avoid red/green combinations to accommodate our color-blind readers).

Source Data files will be made available to reviewers during evaluation of revised manuscripts and, if your paper is eventually published in JCB, the files will be directly linked to specific figures in the published article.

Source Data Figures should be provided as individual PDF files (one file per figure). Authors should endeavor to retain a minimum resolution of 300 dpi or pixels per inch. Please review our instructions for export from Photoshop, Illustrator, and PowerPoint here: https://rupress.org/jcb/pages/submission-guidelines#revised

Journal of Cell Biology now requires a data availability statement for all research article submissions. These statements will be published in the article directly above the Acknowledgments. The statement should address all data underlying the research presented in the manuscript. Please visit the JCB instructions for authors for guidelines and examples of statements at (https://rupress.org/jcb/pages/editorial-policies#data-availability-statement).

B. FINAL FILES:

Please upload the following materials to our online submission system. These items are required prior to acceptance. If you have any questions, contact JCB's Managing Editor, Lindsey Hollander (lhollander@rockefeller.edu).

-- An editable version of the final text (.DOC or .DOCX) is needed for copyediting (no PDFs).

-- High-resolution figure and MP4 video files: See our detailed guidelines for preparing your production-ready images, https://jcb.rupress.org/fig-vid-guidelines.

-- Cover images: If you have any striking images related to this story, we would be happy to consider them for inclusion on the journal cover. Submitted images may also be chosen for highlighting on the journal table of contents or JCB homepage carousel. Images should be uploaded as TIFF or EPS files and must be at least 300 dpi resolution.

It is JCB policy that if requested, original data images must be made available to the editors. Failure to provide original images upon request will result in unavoidable delays in publication. Please ensure that you have access to all original data images prior to final submission.

The license to publish form must be signed before your manuscript can be sent to production. A link to the license to publish form will be sent to the corresponding author only. Please take a moment to check your funder requirements before choosing the appropriate license.

Additionally, JCB encourages authors to submit a short video summary of their work. These videos are intended to convey the main messages of the study to a non-specialist, scientific audience. Think of them as an extended version of your abstract, or a short poster presentation. We encourage first authors to present the results to increase their visibility. The videos will be shared on social media to promote your work. For more detailed guidelines and tips on preparing your video, please visit https://rupress.org/jcb/pages/submission-guidelines#videoSummaries.

Thank you for your attention to these final processing requirements. Please revise and format the manuscript and upload materials within 14 days. If you need an extension for whatever reason, please let us know and we can work with you to determine a suitable revision period.

Please contact the journal office with any questions at cellbio@rockefeller.edu.

Thank you for this interesting contribution, we look forward to publishing your paper in Journal of Cell Biology.

Sincerely,

Sarah Cohen
Monitoring Editor

Andrea L. Marat
Deputy Editor

Journal of Cell Biology

Reviewer #1 (Comments to the Authors (Required)):

The authors have addressed my main concerns. I have only one remaining technical question: although the use of growth curves provides now more convincing support for the differences previously observed on plates, the authors do not specify the number of biological replicates performed. Do the error bars shown in the graphs represent technical replicates?
