# Supplementary material for: Lipid remodeling enables adaptation to chronic hyperosmotic stress
Source: J Cell Biol. 2026 Oct 8;225(11):e202603154. doi: 10.1083/jcb.202603154 (PMC13648197; doi:10.1083/jcb.202603154)
Supplement: Supplementary file 2 [file jcb_202603154_sourcedatafs3.pdf]

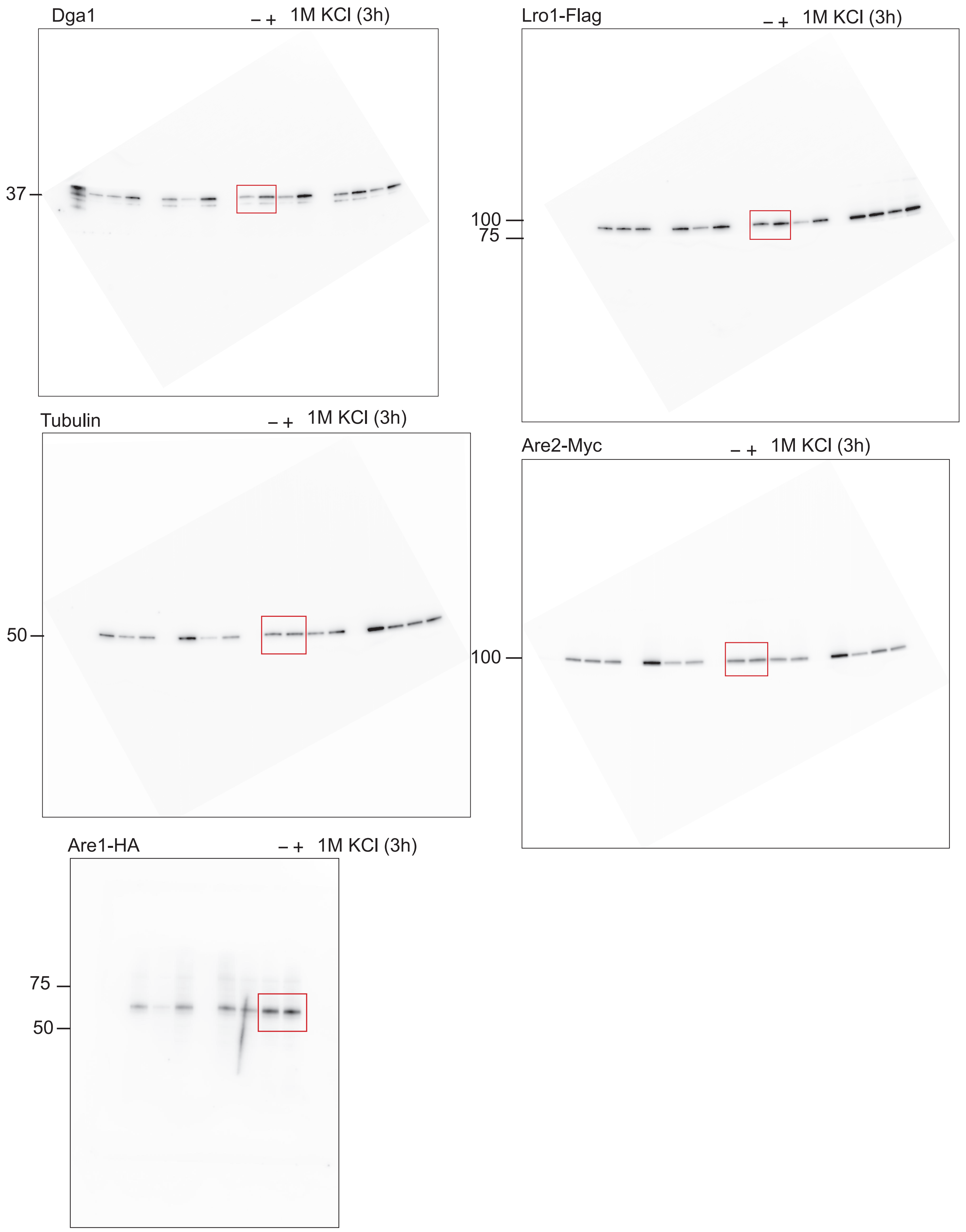
Dga1
− + 1M KCl (3h)
37
Lro1-Flag
− + 1M KCl (3h)
100
75
Tubulin
− + 1M KCl (3h)
50
Are2-Myc
− + 1M KCl (3h)
100
Are1-HA
− + 1M KCl (3h)
75
50
