# Supplementary material for: Lipid remodeling enables adaptation to chronic hyperosmotic stress
Source: J Cell Biol. 2026 Oct 8;225(11):e202603154. doi: 10.1083/jcb.202603154 (PMC13648197; doi:10.1083/jcb.202603154)
Supplement: Supplementary file 3 [file jcb_202603154_sourcedatafs5.pdf]

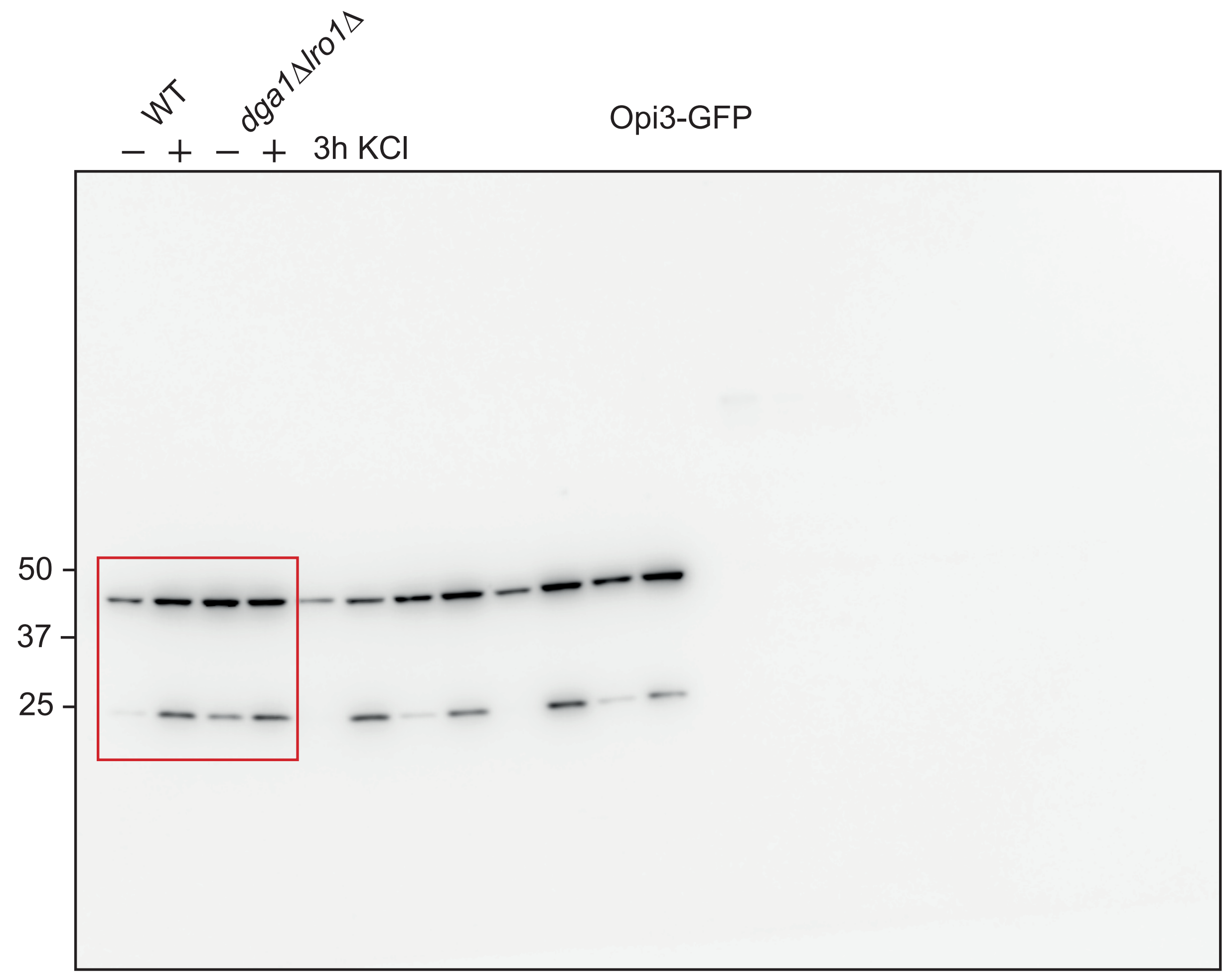
WT
dga1Δlro1Δ
− + − + 3h KCl
Opi3-GFP
50
37
25

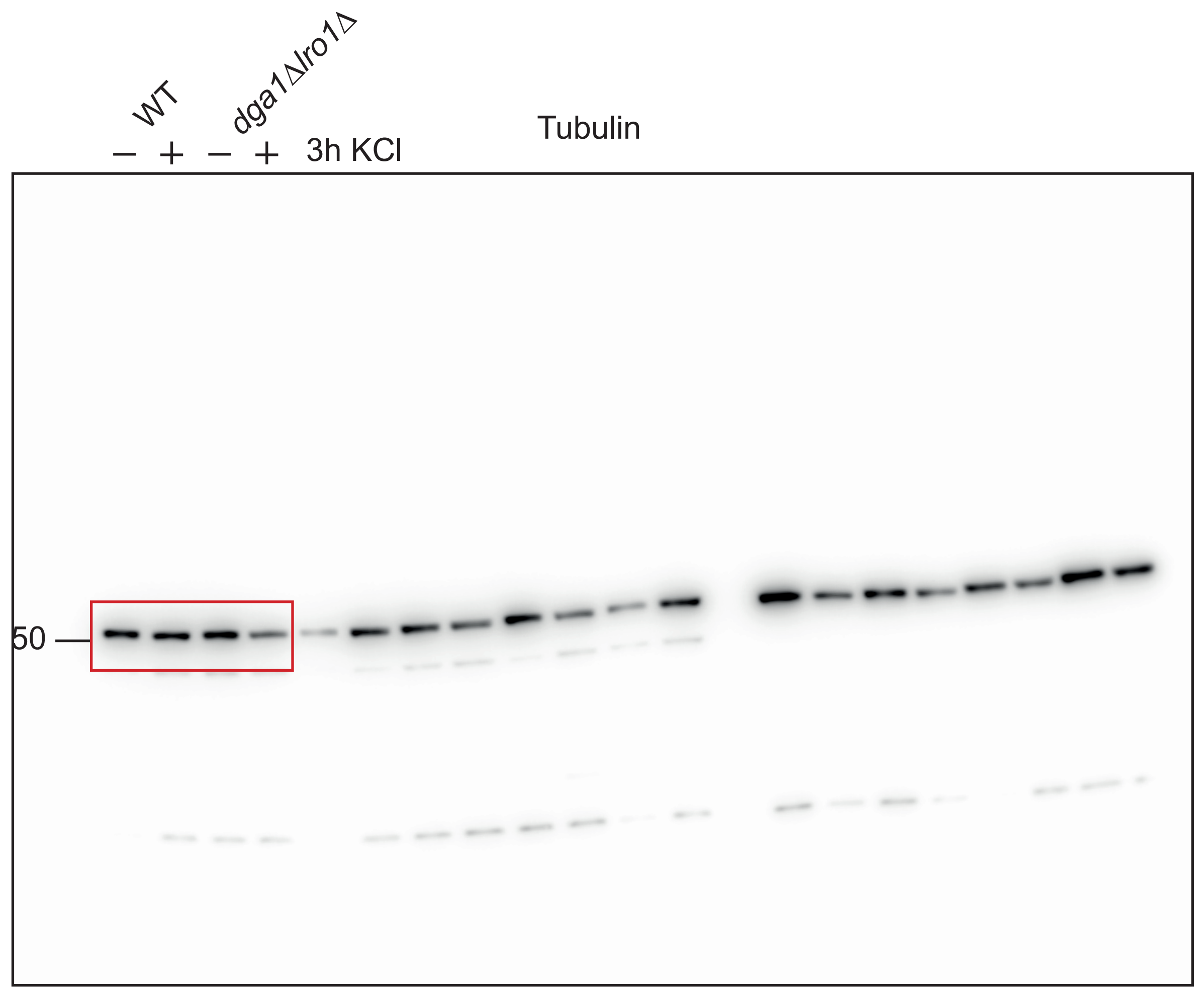
WT
dga1Δlro1Δ
− + − + 3h KCl
Tubulin
50
