# Supplementary material for: Lipid remodeling enables adaptation to chronic hyperosmotic stress
Source: J Cell Biol. 2026 Oct 8;225(11):e202603154. doi: 10.1083/jcb.202603154 (PMC13648197; doi:10.1083/jcb.202603154)
Supplement: Supplementary file 4 [file jcb_202603154_tables1.docx]

**Supplementary Table 1: Strains used in this study.**

*Vectors obtained from [(Williams et al., 2024)](https://sciwheel.com/work/citation?ids=18979175&pre=&suf=&sa=0&dbf=0)

| **Reference** | **Genotype** |
| --- | --- |
| yPC1505 | *MatA ura3∆0 HIS33∆1 leu2∆0 met15∆0* |
| yPC5042 | *MatA ura3∆0 HIS33∆1 leu2∆0 met15∆0 pln1-mCherry::HIS3* |
| yPC13064 | *Matα ura3∆0 HIS33∆1 leu2∆0 met15∆0 pln1-mCherry::HIS3* |
| yPC5450 | *Mat? ura3∆0 HIS33∆1 leu2∆0 met15∆0 sei1∆::NAT pln1-mCherry::HIS3* |
| yPC4929 | *Mat? ura3∆0 HIS33∆1 leu2∆0 met15∆0 are1∆::KAN are2∆::HYG dga1∆::NAT lro1∆::KAN* |
| yPC13088 | *Mat? ura3∆0 HIS33∆1 leu2∆0 met15∆0 lro1∆::KAN pln1-mCherry::HIS3* |
| yPC13552 | *Mat? ura3∆0 HIS33∆1 leu2∆0 met15∆0 lro1∆::KAN pln1-mCherry::HIS3* |
| yPC13538 | *Mat? ura3∆0 HIS33∆1 leu2∆0 met15∆0 cho2∆::NAT psd1∆::HYG psd2∆::KAN pln1-mCherry::HIS3* |
| yPC13539 | *Mat? ura3∆0 HIS33∆1 leu2∆0 met15∆0 cho2∆::NAT psd1∆::HYG psd2∆::KAN pln1-mCherry::HIS3* |
| yPC13082 | *Mat? ura3∆0 HIS33∆1 leu2∆0 met15∆0 are1∆::KAN are2∆::HYG pln1-mCherry::HIS3* |
| yPC13085 | *MatA ura3∆0 HIS33∆1 leu2∆0 met15∆0 dga1∆::NAT lro1∆::KAN pln1-mCherry::HIS3* |
| yPC13518 | *Matα ura3∆0 HIS33∆1 leu2∆0 met15∆0 dga1∆::NAT lro1∆::KAN pln1-mCherry::HIS3* |
| yPC13565 | *Mat? ura3∆0 HIS33∆1 leu2∆0 met15∆0 dga1∆::NAT pln1-mCherry::HIS3* |
| yPC13566 | *Mat? ura3∆0 HIS33∆1 leu2∆0 met15∆0 dga1∆::NAT pln1-mCherry::HIS3* |
| yPC13356 | *Mat? ura3∆0 HIS33∆1 leu2∆0 met15∆0 are1-HA::HIS3 are2-MYC::KAN lro1-FLAG::HYG* |
| yPC10975 | *MatA ura3∆0 HIS33∆1 leu2∆0 met15∆0 dga1-mNeonGreen::HIS3* |
| yPC13068 | *Mat? ura3∆0 HIS33∆1 leu2∆0 met15∆0 nem1∆::KAN pln1-mCherry::HIS3* |
| yPC13069 | *Mat? ura3∆0 HIS33∆1 leu2∆0 met15∆0 nem1∆::KAN pln1-mCherry::HIS3* |
| yPC13342 | *MatA ura3∆0 HIS33∆1 leu2∆0 met15∆0 faa1∆::KAN pln1-mCherry::HIS3* |
| yPC13343 | *MatA ura3∆0 HIS33∆1 leu2∆0 met15∆0 faa1∆::KAN pln1-mCherry::HIS3* |
| yPC13496 | *Mat? ura3∆0 HIS33∆1 leu2∆0 met15∆0 faa4∆::NAT pln1-mCherry::HIS3* |
| yPC13497 | *Mat? ura3∆0 HIS33∆1 leu2∆0 met15∆0 faa4∆::NAT pln1-mCherry::HIS3* |
| yPC13376 | *Mat? ura3∆0 HIS33∆1 leu2∆0 met15∆0 faa1∆::KAN faa4∆::NAT pln1-mCherry::HIS3* |
| yPC13377 | *Mat? ura3∆0 HIS33∆1 leu2∆0 met15∆0 faa1∆::KAN faa4∆::NAT pln1-mCherry::HIS3* |
| yPC5609 | *Matα ura3∆0 HIS33∆1 leu2∆0 met15∆0 lro1-mCherry::HIS3* |
| yPC6703 | *MatA ura3∆0 HIS33∆1 leu2∆0 met15∆0 are1-GFP::HIS5* |
| yPC5608 | *Matα ura3∆0 HIS33∆1 leu2∆0 met15∆0 are2-mCherry::URA3* |
| yPC13120 | *Mat? ura3∆0 HIS33∆1 leu2∆0 met15∆0 hog1∆::KAN pln1-mCherry::HIS3* |
| yPC13121 | *Mat? ura3∆0 HIS33∆1 leu2∆0 met15∆0 hog1∆::KAN pln1-mCherry::HIS3* |
| yPC13206 | *Mat? ura3∆0 HIS33∆1 leu2∆0 met15∆0 hog1∆::URA3 pln1-mCherry::HIS3* |
| yPC13196 | *Mat? ura3∆0 HIS33∆1 leu2∆0 met15∆0 gpd1∆::KAN pln1-mCherry::HIS3* |
| yPC13197 | *Mat? ura3∆0 HIS33∆1 leu2∆0 met15∆0 gpd1∆::KAN pln1-mCherry::HIS3* |
| yPC13245 | *Mat? ura3∆0 HIS33∆1 leu2∆0 met15∆0 hog1∆::URA3 dga1∆::NAT lro1∆::KAN pln1-mCherry::HIS3* |
| yPC13249 | *Mat? ura3∆0 HIS33∆1 leu2∆0 met15∆0 hog1∆::URA3 dga1∆::NAT lro1∆::KAN pln1-mCherry::HIS3* |
| yPC13244 | *Mat? ura3∆0 HIS33∆1 leu2∆0 met15∆0 hog1∆::URA3 dga1∆::NAT pln1-mCherry::HIS3* |
| yPC13247 | *Mat? ura3∆0 HIS33∆1 leu2∆0 met15∆0 hog1∆::URA3 dga1∆::NAT pln1-mCherry::HIS3* |
| yPC13246 | *Mat? ura3∆0 HIS33∆1 leu2∆0 met15∆0 hog1∆::URA3 lro1∆::KAN pln1-mCherry::HIS3* |
| yPC13248 | *Mat? ura3∆0 HIS33∆1 leu2∆0 met15∆0 hog1∆::URA3 lro1∆::KAN pln1-mCherry::HIS3* |
| yPC13203 | *Mat? ura3∆0 HIS33∆1 leu2∆0 met15∆0 sln1-DAmP::KAN pln1-mCherry::HIS3* |
| yPC13163 | *Mat? ura3∆0 HIS33∆1 leu2∆0 met15∆0 ptc1∆::KAN pln1-mCherry::HIS3* |
| yPC13166 | *Mat? ura3∆0 HIS33∆1 leu2∆0 met15∆0 ptc1∆::KAN pln1-mCherry::HIS3* |
| yPC13227 | *Mat? ura3∆0 HIS33∆1 leu2∆0 met15∆0 pbs2∆::KAN pln1-mCherry::HIS3* |
| yPC13256 | *Mat? ura3∆0 HIS33∆1 leu2∆0 met15∆0 sho1∆::HYG ssk1∆::KAN pln1-mCherry::HIS3* |
| yPC13228 | *Mat? ura3∆0 HIS33∆1 leu2∆0 met15∆0 pbs2∆::KAN pln1-mCherry::HIS3* |
| yPC13257 | *Mat? ura3∆0 HIS33∆1 leu2∆0 met15∆0 sho1∆::HYG ssk1∆::KAN pln1-mCherry::HIS3* |
| yPC13281 | *Mat? ura3∆0 HIS33∆1 leu2∆0 met15∆0 hkr1∆::KAN msb2∆::NAT pln1-mCherry::HIS3* |
| yPC13282 | *Mat? ura3∆0 HIS33∆1 leu2∆0 met15∆0 hkr1∆::KAN msb2∆::NAT pln1-mCherry::HIS3* |
| yPC13289 | *Mat? ura3∆0 HIS33∆1 leu2∆0 met15∆0 hkr1∆::KAN ypd1-DAmP::NAT pln1-mCherry::HIS3* |
| yPC13290 | *Mat? ura3∆0 HIS33∆1 leu2∆0 met15∆0 hkr1∆::KAN ypd1-DAmP::NAT pln1-mCherry::HIS3* |
| yPC13287 | *Mat? ura3∆0 HIS33∆1 leu2∆0 met15∆0 ypd1-DAmP::KAN msb2∆::NAT pln1-mCherry::HIS3* |
| yPC13288 | *Mat? ura3∆0 HIS33∆1 leu2∆0 met15∆0 ypd1-DAmP::KAN msb2∆::NAT pln1-mCherry::HIS3* |
| yPC13428 | *Mat? ura3∆0 HIS33∆1 leu2∆0 met15∆0 ede1∆::NAT pln1-mCherry::HIS3* |
| yPC13429 | *Mat? ura3∆0 HIS33∆1 leu2∆0 met15∆0 ede1∆::NAT pln1-mCherry::HIS3* |
| yPC13428 | *Mat? ura3∆0 HIS33∆1 leu2∆0 met15∆0 ede1∆::NAT pln1-mCherry::HIS3* |
| yPC13418 | *Mat? ura3∆0 HIS33∆1 leu2∆0 met15∆0 osh2∆::NAT osh3∆::KAN pln1-mCherry::HIS3* |
| yPC13419 | *Mat? ura3∆0 HIS33∆1 leu2∆0 met15∆0 osh2∆::NAT osh3∆::KAN pln1-mCherry::HIS3* |
| yPC13113 | *MatA ura3∆0 HIS33∆1 leu2∆0 met15∆0 hog1∆::KAN* |
| yPC13246 | *Mat? ura3∆0 HIS33∆1 leu2∆0 met15∆0 hog1∆::URA3 lro1∆::KAN pln1-mCherry::HIS3* |
| yPC13248 | *Mat? ura3∆0 HIS33∆1 leu2∆0 met15∆0 hog1∆::URA3 lro1∆::KAN pln1-mCherry::HIS3* |
| yPC13244 | *Mat? ura3∆0 HIS33∆1 leu2∆0 met15∆0 hog1∆::URA3 dga1∆::NAT pln1-mCherry::HIS3* |
| yPC13247 | *Mat? ura3∆0 HIS33∆1 leu2∆0 met15∆0 hog1∆::URA3 dga1∆::NAT pln1-mCherry::HIS3* |
| yPC13245 | *Mat? ura3∆0 HIS33∆1 leu2∆0 met15∆0 hog1∆::URA3 dga1∆::NAT lro1∆::KAN pln1-mCherry::HIS3* |
| yPC13249 | *Mat? ura3∆0 HIS33∆1 leu2∆0 met15∆0 hog1∆::URA3 dga1∆::NAT lro1∆::KAN pln1-mCherry::HIS3* |
| yPC5042 + pTW114* | *MatA ura3∆0 HIS33∆1 leu2∆0 met15∆0 pln1-mCherry::HIS3 + GPDpr-Sch9-3xHA::URA3* |
| yPC5042 + pTW123* | *MatA ura3∆0 HIS33∆1 leu2∆0 met15∆0 pln1-mCherry::HIS3 + GPDpr-Sch9-2D3E-3xHA::URA3* |
| yPC13292 | *MatA ura3∆0 HIS33∆1 leu2∆0 met15∆0 sit4∆::KAN pln1-mCherry::HIS3* |
| yPC13293 | *MatA ura3∆0 HIS33∆1 leu2∆0 met15∆0 sit4∆::KAN pln1-mCherry::HIS3* |
| yPC13072 | *Mat? ura3∆0 HIS33∆1 leu2∆0 met15∆0 mpk1∆::KAN pln1-mCherry::HIS3* |
| yPC13073 | *Mat? ura3∆0 HIS33∆1 leu2∆0 met15∆0 mpk1∆::KAN pln1-mCherry::HIS3* |
| yPC13283 | *Mat? ura3∆0 HIS33∆1 leu2∆0 met15∆0 hac1∆::KAN pln1-mCherry::HIS3* |
| yPC13284 | *Mat? ura3∆0 HIS33∆1 leu2∆0 met15∆0 hac1∆::KAN pln1-mCherry::HIS3* |
| yPC13553 | *Mat? ura3∆0 HIS33∆1 leu2∆0 met15∆0 pct1∆::HYG dga1∆::NAT lro1∆::KAN pln1-mCherry::HIS3* |
| yPC13554 | *Mat? ura3∆0 HIS33∆1 leu2∆0 met15∆0 pct1∆::HYG dga1∆::NAT lro1∆::KAN pln1-mCherry::HIS3* |
| yPC1573 | *MatA ura3∆0 HIS33∆1 leu2∆0 met15∆0 sec63-GFP::HIS5* |
| yPC13582 | *MatA ura3∆0 HIS33∆1 leu2∆0 met15∆0 dga1∆::NAT lro1∆::KAN sec63-GFP::HIS5* |
| yPC13584 | *MatA ura3∆0 HIS33∆1 leu2∆0 met15∆0 ilv6-GFP::HIS5* |
| yPC13590 | *MatA ura3∆0 HIS33∆1 leu2∆0 met15∆0 dga1∆::NAT lro1∆::KAN sec63-GFP::HIS5* |
| yPC6692 | *MatA ura3∆0 HIS33∆1 leu2∆0 met15∆0 cho2-GFP::HIS5* |
| yPC5926 | *MatA ura3∆0 HIS33∆1 leu2∆0 met15∆0 opi3-GFP::HIS5* |
| yPC13569 | *Mat? ura3∆0 HIS33∆1 leu2∆0 met15∆0 dga1∆::NAT lro1∆::KAN cho2-GFP::HIS5* |
| yPC13570 | *Mat? ura3∆0 HIS33∆1 leu2∆0 met15∆0 dga1∆::NAT lro1∆::KAN cho2-GFP::HIS5* |
| yPC13573 | *Mat? ura3∆0 HIS33∆1 leu2∆0 met15∆0 dga1∆::NAT lro1∆::KAN opi3-GFP::HIS5* |
