# Supplementary material for: Lipid remodeling enables adaptation to chronic hyperosmotic stress
Source: J Cell Biol. 2026 Oct 8;225(11):e202603154. doi: 10.1083/jcb.202603154 (PMC13648197; doi:10.1083/jcb.202603154)
Supplement: Supplementary file 5 [file jcb_202603154_tables2.docx]

**Supplementary Table 2: Primers used in this study.**

| **Name** | **Target mRNA** | **Sequence** |
| --- | --- | --- |
| oTWPC166 | *TUB2* | ATGGCCACCTTCTCCGTCTT |
| oTWPC167 | *TUB2* | AGACATGACGCTCGAGACCA |
| oTWPC168 | *DGA1* | GGAAGCCCTACAGCCGGTAT |
| oTWPC169 | *DGA1* | GTGCTGGTGTCGAGATTGCA |
| oTWPC176 | *LRO1* | TGCTGCGTATGATTGGAGGC |
| oTWPC177 | *LRO1* | CAGCCACGACCACCATTACC |
| oTWPC178 | *ARE1* | CCTCATGATGGTCACGGCAC |
| oTWPC179 | *ARE1* | ACTCCGCCACGCAATTCAAT |
| oTWPC180 | *ARE2* | GCACGGTATAGGCAAGGGTC |
| oTWPC181 | *ARE2* | GGAGGACTTGCGGTGGTGTA |
| oTWPC170 | *CHO2* | GCGTTGCAACAAGATTGAGTCC |
| oTWPC171 | *CHO2* | GTCGCACTCCAATGACCACC |
| oTWPC172 | *OPI3* | GTGTTCTCTGATCACGGGCG |
| oTWPC173 | *OPI3* | AAACGTTGAAGGGGAAGCCG |
